# A diverse Ediacara assemblage survived under low-oxygen conditions

**DOI:** 10.1038/s41467-022-35012-y

**Published:** 2022-11-27

**Authors:** Lucas B. Cherry, Geoffrey J. Gilleaudeau, Dmitriy V. Grazhdankin, Stephen J. Romaniello, Aaron J. Martin, Alan J. Kaufman

**Affiliations:** 1 Department of Atmospheric, Oceanic, and Earth Sciences, George Mason University, Fairfax, VA USA; 2 Department of Geology, University of Maryland, College Park, MD USA; 3 Trofimuk Institute of Petroleum Geology and Geophysics, Russian Academy of Sciences, Siberian Branch, Novosibirsk Russia; 4 School of Earth and Space Exploration, Arizona State University, Tempe, AZ USA; 5 Division de Geociencias Aplicadas, IPICYT, CP 78216, San Luis Potosí San Luis Potosí, Mexico; 6 Department of Geology and Earth System Science Interdisciplinary Center, University of Maryland, College Park, MD USA

**Keywords:** Element cycles, Marine chemistry

## Abstract

The Ediacaran biota were soft-bodied organisms, many with enigmatic phylogenetic placement and ecology, living in marine environments between 574 and 539 million years ago. Some studies hypothesize a metazoan affinity and aerobic metabolism for these taxa, whereas others propose a fundamentally separate taxonomic grouping and a reliance on chemoautotrophy. To distinguish between these hypotheses and test the redox-sensitivity of Ediacaran organisms, here we present a high-resolution local and global redox dataset from carbonates that contain in situ Ediacaran fossils from Siberia. Cerium anomalies are consistently >1, indicating that local environments, where a diverse Ediacaran assemblage is preserved in situ as nodules and carbonaceous compressions, were pervasively anoxic. Additionally, δ^238^U values match other terminal Ediacaran sections, indicating widespread marine euxinia. These data suggest that some Ediacaran biotas were tolerant of at least intermittent anoxia, and thus had the capacity for a facultatively anaerobic lifestyle. Alternatively, these soft-bodied Ediacara organisms may have colonized the seafloor during brief oxygenation events not recorded by redox proxy data. Broad temporal correlations between carbon, sulfur, and uranium isotopes further highlight the dynamic redox landscape of Ediacaran-Cambrian evolutionary events.

## Abstract (web summary)

Geochemical data from sedimentary rocks in Siberia indicate that members of the soft-bodied Ediacara biota (the earliest macroscopic life on Earth) were tolerant of low-oxygen conditions, suggesting they had the capacity for anaerobic metabolisms.

## Introduction

Of the enigmatic Ediacaran biotas—the earliest macroscopic organisms on Earth that persisted for about 35 million years (ca. 574-539 Ma) in globally distributed marine environments—the Rangeomorphs, characterized by their modular organic-walled fronds and fractal level of organization, are considered by some to represent stem group eumetazoans^1^. However, because of their unusual moldic preservation in sandstones (or as nodules or carbonaceous compressions in carbonates) and bizarre quilted morphologies, the metabolic requirements of these organisms remain controversial. Given the extreme surface area of the fronds, which preserve similar patterns at increasingly smaller scales^2,3^, recent interpretations of Rangeomorph feeding strategies range from osmotrophy (absorption of labile dissolved organic carbon from seawater), to chemosymbiosis (with autotrophic sulfur-oxidizing bacteria), to extracellular digestion within semi-isolated chambers (including a resident microbiome)^4–10^. Other hypotheses suggest an aerobic metabolism for the Rangeomorphs^11,12^, leading to the hypothesis that a rise in the oxidation state of seawater along continental shelves was the primary controlling factor in their evolution, and its fall to their extinction^13^. To the contrary, if the Rangeomorphs did not require oxygen for their metabolism, their evolution may have been independent of ocean redox conditions.

Distinguishing between these two hypotheses is critical to understanding the biological affinity of the Ediacaran biotas, and requires constraints on the redox conditions under which they lived. Emerging evidence suggests a highly dynamic oxidation state through Ediacaran space and time. Based on geochemical evidence, the geological period may be divided into discrete intervals with different proportions of oxic^13–16^ and anoxic seafloor conditions^17,18^. Furthermore, data support the presence of redox gradients across facies on individual platforms, suggesting that the regional distribution of Ediacaran organisms was dependent on the oxidation state of overlying seawater^19–23^. Globally, most of the fossils of soft-bodied organisms are preserved as casts and molds in medium- to coarse-grained sandstones—which are not amenable to paleo-redox reconstruction—rather than in fine-grained shales, which do contain distributions of redox-sensitive elements that may be used to reconstruct environmental conditions^24^. In rare and fortuitous instances, Ediacaran biotas have been preserved as nodules and compressions in carbonates, for which proxies for both local and global redox conditions have been calibrated. Here, we present high-resolution time-series data for both local and global ocean redox in carbonates from arctic Siberia that also contain Rangeomorphs, along with a numerically-abundant assemblage of macrofossils consisting of Arboreomorphs (frondose organisms with holdfasts rooted in substrate), undifferentiated bulbous structures with tubules reaching into surrounding sediments, and large carbonaceous compressions.

**Fig. 1:**
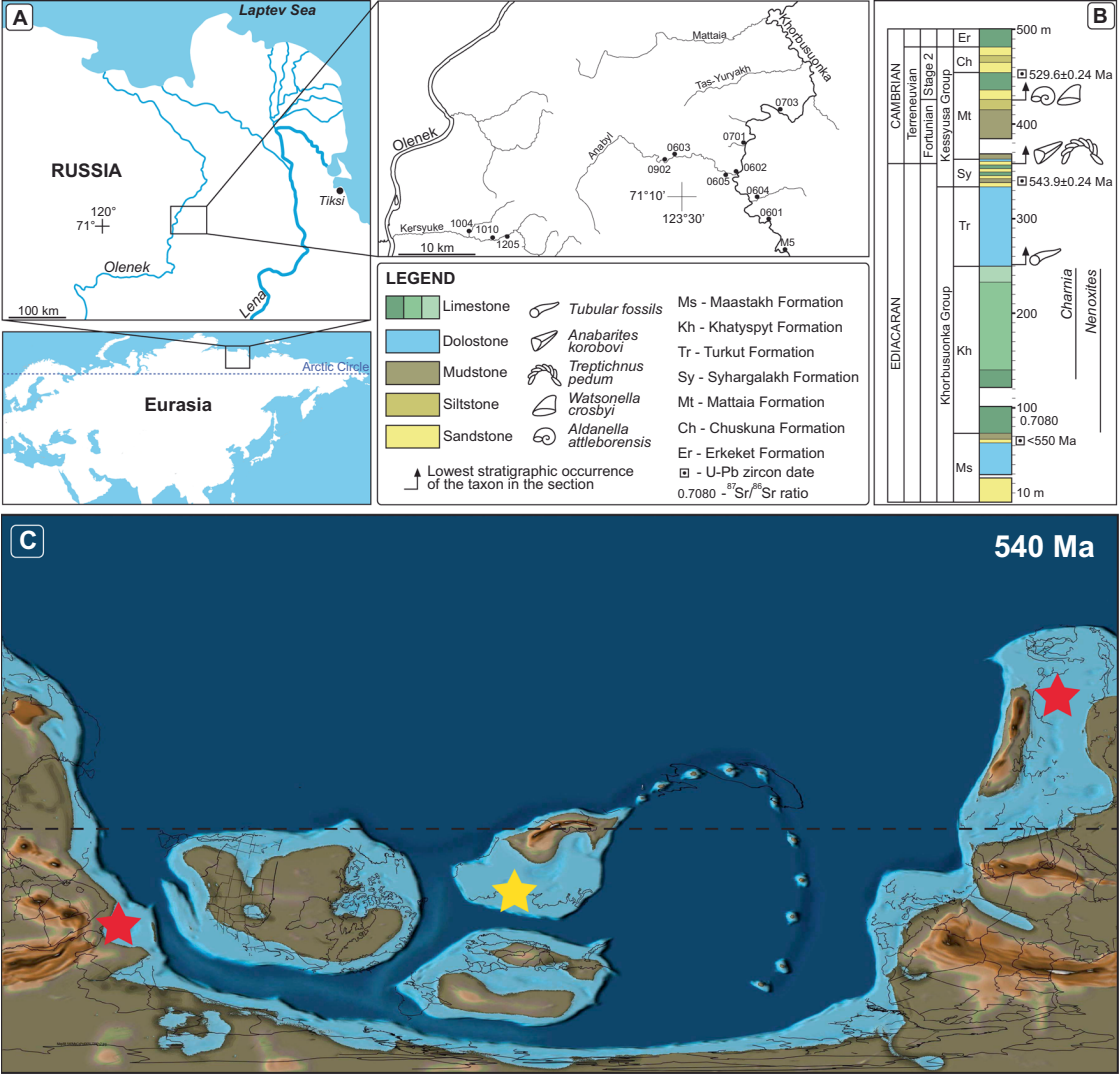
Geologic and paleogeographic background of the Olenek Uplift. **A** Map of the study area and measured sections along the Khorbusuonka and Olenek River areas of northeastern Siberia. **B** Generalized stratigraphic column of the study units including lithology, fossil content, and radiometric age constraints. ^87^Sr/^86^Sr value from ref. 8 and radiometric ages from refs. 30, 33, and this study. Maps in panels **A** and **B** were created by the authors using: www.yandex.ru/maps. **C** Paleogeographic map from 540 Ma with the Olenek Uplift section examined in this study denoted by the yellow star and the coeval Namibia and South China sections denoted by the red stars. Base map from, ref. 102: Scotese, C.R., 2021. An Atlas of Paleographic Maps: The Seas Come In and the Seas Go Out, Annual Reviews of Earth and Planetary Sciences, v. 49, p. 669–718.

The Olenek Uplift region of northeastern Siberia contains a well-studied succession of terminal Ediacaran to lower Cambrian strata, including five units of primary interest: (1) the Maastakh Formation, consisting of partially silicified peritidal dolostone with chert nodules, cross-bedding, and wave ripples; (2) the fossiliferous Khatyspyt Formation, consisting of thin- to medium-bedded bituminous limestone; (3) the Turkut Formation, comprising dolostone and limestone with several levels of collapse breccia filled with pyrobitumen that may be related to evaporite dissolution; (4) siliciclastics and volcanics of the Syhargalakh Formation; and (5) mixed siliciclastics and allodapic limestones of the Mattaia Formation^25–28^ (Fig. 1).

Detrital zircon U-Pb isotopic ages provide a maximum depositional age of 550 Ma for a sandstone near the top of the Maastakh Formation (see Supplementary Discussion; Supplementary Figs. 11, 12). This age is relevant because it constrains the overlying Khatyspyt Formation as younger than 550 Ma, which resolves an important uncertainty in late Ediacaran chronostratigraphy^29^. Specifically, ^87^Sr/^86^Sr values for the Khatyspyt Formation are lower (near 0.7080; ref. 8) than other late Ediacaran successions from China, Oman, and Australia deposited during and after the Shuram excursion, which left an uncertainty as to whether the Khatyspyt Formation was older or younger that those successions. Our U-Pb age resolves this issue and places the Khatyspyt Formation in the terminal Ediacaran Period, younger than 550 Ma. A terminal Ediacaran age is supported for the Turkut Formation and underlying units by a U-Pb zircon date of 543.9 ± 0.24 Ma generated for a tuff breccia within the Syhargalakh Formation^30^. While this date was originally thought to provide a constraint on the Ediacaran-Cambrian boundary, a U-Pb zircon age of ca. 538.8 Ma from Namibia provides a new radiometric marker for this critical transition^31^, and refs. 29, 32. further suggested that the Ediacaran-Cambrian boundary may be <538 Ma based on the presence of Ediacara-type fossils up to an ash bed dated to 538.3 ± 0.14 Ma in South Africa and the inference that the base of the Cambrian Period (defined bio- and chemostratigraphically) must be younger than this horizon. A second U-Pb zircon date of 529.56 ± 0.24 Ma in a volcanic ash immediately overlying the Mattaia carbonate platform^33^ constrains the age of small shelly fossils (SSFs) of the *N. sunnaginacus* Zone and thereby the onset of the Cambrian explosion^28^.

The Olenek Uplift is known for containing well-preserved nodules and carbonaceous compressions of Ediacaran organisms, including the first preserved appearance of *Nenoxites curvus* and a diverse Ediacara assemblage of soft-bodied organisms in the Khatyspyt Formation (Supplementary Figs. 1 and 2), which are morphologically and environmentally similar to Avalon-type fossils from Newfoundland^34^. The Turkut Formation contains the first appearance of tubular steinkerns, which are preserved as smooth-walled molds. While the first SSFs of Cambrian aspect (*Anabarites trisulcatus* assemblage Zone) appear near the base of the Syhargalakh Formation, the lowest occurrence of *Treptichnius pedum* (the ichnospecies associated with the Ediacaran-Cambrian boundary) and *Rusophycus* (a likely arthropod resting trace) are found at the top of the Syhargalakh Formation. Stratigraphically higher in the Mattaia Formation, more complex traces of organisms that burrowed deeply into the sediments, as well as transported SSFs of the lower Cambrian Fortunian Stage *Purella antiqua* and Cambrian Stage 2 *Nochoroicyathus sunnaginicus* assemblage zones, are found in siliciclastic and carbonate facies^28^.

**Fig. 2:**
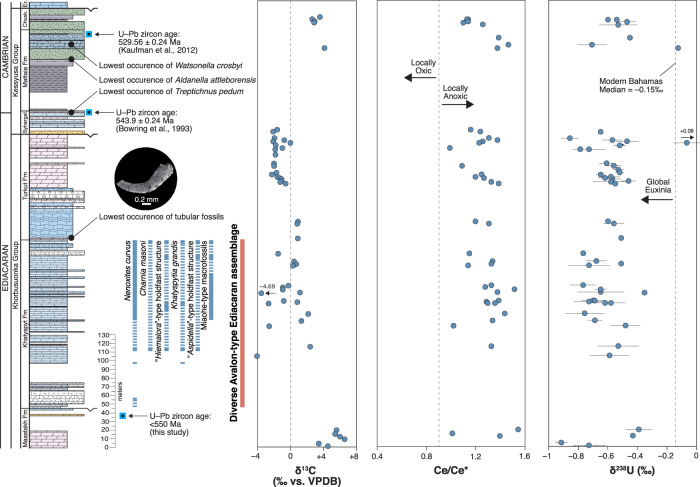
Olenek Uplift chemostratigraphy. Stratigraphic column, fossil occurrences, and geochemical data (δ^13^C, δ^238^U, and Ce/Ce*) for our composite Olenek Uplift section. δ^13^C data are from ref. 99. Paleontology is described in detail in ref. 34. Modern Bahamas median δ^238^U value is from ref. 55 and oxic/anoxic Ce/Ce* cutoff value of 0.9 is from ref. 21. Note that the U-Pb zircon age of 543.9 ± 0.24 Ma published by ref. 30 from the Syhargalakh Formation was reinterpreted as 542.8 ± 1.3 Ma using the most concordant grains by ref. 103.

Carbon isotopes (δ^13^C) are used to correlate Olenek Uplift strata with other terminal Ediacaran and basal Cambrian successions worldwide, in particular to coeval intervals in South China (see ref. 29 for review). In arctic Siberia, time-series δ^13^C values show a decreasing trend from near +6‰ in the Maastakh and basal Khatyspyt formations, and then an oscillation between −5 and +4‰ through the overlying reaches of the interval^25,26,35^ before stabilizing near 0‰ in dolostones of the lower Turkut Formation. In the upper levels of the Turkut Formation, δ^13^C values are noted to stratigraphically decline to as low as −4‰ before the carbonate platform is regionally truncated beneath a disconformity surface. Carbonates in the overlying Mattaia Formation preserve a negative-to-positive trend, ending in peak values as high as +5‰ (Fig. 2) associated with SSFs of the *N. sunnaginicus* assemblage Zone marking the base of Cambrian Stage 2^28^. Correlation to South China suggests that the strongly positive δ^13^C values in the Maastakh Formation are equivalent to similarly high values in the basal Dengying Formation (Beiwan Member^36^), while a second positive carbon isotope peak in the Gaojiashan Member associated with the first appearance of *Cloudina* in South China^35^ most likely equates to a similar magnitude excursion in the Khatyspyt Formation. The negative δ^13^C excursion seen across the Ediacaran-Cambrian boundary (BACE event^37^) beginning at the top of the Dengying Formation in South China (where values drop to as low as −7‰) may begin in the upper Turkut Formation^25,26^, but the sedimentary rocks preserving the negative carbon isotope anomaly are regionally truncated at an unconformity surface below siliciclastics of the Syhargalakh Formation. Alternatively, the shift towards negative δ^13^C values in the upper Turkut Formation could correlate with the A4 anomaly, which is a negative δ^13^C excursion identified as older than the BACE event in Oman^29,32^. Higher in the succession, peak δ^13^C values of the Mattaia carbonate platform are potentially equivalent to those in the Zhujiaqiang Formation of South China, which are also biostratigraphically constrained to Cambrian Stage 2 based on the first appearance of *N. sunnaginacus* assemblage Zone fossils.

The time-series trend in sulfur isotope (δ^34^S) compositions of pyrite in acidified residues from Khatyspyt limestones may further provide a chemostratigraphic marker of terminal Ediacaran time. The stratigraphic data show a profound shift from −20 to +30‰ that has been interpreted as a change in redox conditions and/or sulfate availability on a regional or global scale^20^. Notably, the first appearance of soft-bodied Ediacara biotas in the Khatyspyt Formation and a positive δ^13^C excursion are associated with this prominent perturbation in seawater chemistry. A remarkably similar sulfur isotope anomaly is recorded in the Gaojiashan Member of the Dengying Formation, which is associated with the upper of the two positive carbon isotope excursions in the formation, and the first evidence for metazoan biomineralization in South China^35^. Examples of Ediacaran ‘superheavy pyrite’ in the aftermath of the Shuram Excursion are also known from Oman^38^, Namibia^39^, northwestern Canada^40^, and northwestern China^41^. Some Ediacaran ‘superheavy pyrite’ values have been attributed to anomalies in laboratory extraction methods^42^ or thermochemical sulfate reduction within hydrothermal fluids^43^. However, their coincidence specifically in post-Shuram successions worldwide supports the view that these events can be used as chemostratigraphic markers, and most-likely reflect significant changes in the redox landscape of the global ocean.

Here we investigate local redox conditions in both limestones and dolostones of the Olenek Uplift succession using the magnitude of cerium (Ce) anomalies. Cerium is a redox-sensitive rare earth element (REE) where Ce(III) can be partially oxidized to Ce(IV) under oxic conditions. This causes a decrease in solubility, which results in Ce removal from seawater. Modern well-oxygenated seawater is thus depleted in Ce relative to other trivalent rare-earth elements (REEs), resulting in a pronounced negative Ce anomaly (Ce/Ce* = [Ce]/([Pr]^2^/[Nd]) < 1 with element concentrations normalized to the post-Archean Australian shale)^44^. In contrast, Ce stays in solution under anoxic conditions and anoxic waters have little to no Ce anomaly, as evidenced by Ce/Ce* values that are close to or >1 below the chemocline in many modern anoxic basins^45^. Cerium oxidation is closely linked to the redox kinetics of manganese (Mn) such that the presence/absence of a Ce anomaly is specifically sensitive to the position of a sample with respect to the Mn redoxcline^45^. In the suboxic portion of the modern Black Sea, Mn is actively oxidized at O_2_ concentrations below analytical detection (<3 μM)^46^, which helps constrain the threshold needed to generate a negative Ce anomaly. Marine carbonates can preserve a reliable record of seawater REE patterns—as a substitution for Ca^2+^ in carbonate minerals^47^—if diagenetic alteration and detrital contamination are minimized^48^, with a recent study of modern Bahamian carbonates indicating that REE signals are particularly resistant to diagenesis^49^. Samples can be screened for detrital influence using yttrium to holmium (Y/Ho) ratios, with values >36 indicating an authigenic seawater signal^48^.

We also estimate global seawater redox conditions, in large part related to the extent of euxinic (free H_2_S in the water column) conditions, using uranium (U) isotopes. Uranium in nature is dominated by two long-lived isotopes, ^235^U and ^238^U, whose half-lives are 0.7 and 4.5 Ga, respectively. In nature, U exists in predominantly two oxidation states: soluble U(VI) and insoluble, particle-reactive U(IV). Soluble U(VI) forms unreactive, stable calcium-uranyl-bicarbonate complexes in seawater. It is well-mixed and isotopically homogenous in the modern ocean with an isotopic composition of −0.39‰^50^. Among various sinks for U in the ocean, the strongest isotopic fractionation is associated with removal of U to euxinic sediments, which preferentially sequester isotopically-heavy ^238^U, thereby leaving residual seawater more enriched in isotopically light ^235^U. Thus, an expansion of ocean euxinia will drive the δ^238^U composition of global seawater towards lower values. Recent data suggest that substantially less fractionation occurs under ferruginous (free Fe^2+^ in the water column) conditions^51^ and suboxic conditions^52^ than under euxinic conditions. Thus, U isotopes are increasingly considered a proxy specifically for marine euxinia^52,53^. Reference 54 also found a relationship between the degree of isotopic fractionation imparted during U removal to anoxic sediments and hydrographic factors such as primary productivity and basin restriction. Despite these complications, the size of the euxinic sink for U in the oceans clearly exerts a primary control on seawater δ^238^U values. The δ^238^U signal of seawater can faithfully be recorded in marine carbonate sediments under certain conditions, with modern Bahamian carbonates showing a small but consistent isotopic offset between seawater and sediment regardless of burial depth and mineralogy (Bahamas median = −0.15‰ vs. seawater = −0.39‰)^55^. Taken together, a shift towards lighter U isotopes in ancient carbonates can be interpreted as an expansion of global marine euxinia.

Ultimately, this study combines proxies for local (Ce anomaly) and global (U isotope) redox conditions in the same carbonate strata that preserve in situ Ediacaran fossils, thus allowing us to examine the redox context of the enigmatic biota. The occurrence of well-preserved Ediacaran fossils in carbonate environments allows us to directly link in situ oxidation state, global ocean redox, and fossil assemblages in the same strata, thus providing important constraints on the redox-dependence and metabolism of early macroscopic life on Earth.

## Results

### Diagenesis

We initially assessed the diagenetic grade of our 56 selected Olenek Uplift carbonates using petrographic observations and typical geochemical tracers such as the manganese to strontium (Mn/Sr) ratios and oxygen isotope (δ^18^O) compositions. Petrographic analysis (Supplementary Figs. 3–6) of most samples revealed a fine-grained micritic to microsparitic matrix with the presence of fine, organic-rich laminations in the Khatyspyt Formation, as well as micritic carbonates associated with transported skeletal fossils in the Mattaia Formation. In contrast, the Turkut Formation contains fine-grained dolomicrite and dolomicrosparite in most intervals, with occasional horizons of coarse interlocking dolomite spar that indicate fabric-destructive recrystallization. The Mn/Sr ratio (less than the typical cutoff value of 10; ref. 56) also indicates a high degree of preservation for the sample set, with the exception of the coarser dolomites from the upper Turkut Formation. The range of δ^18^O values (between −0.65 and −8.38‰) are in a reasonable range for well-preserved Proterozoic carbonates^57^ with the heaviest values in the Turkut Formation consistent with the presence of collapse breccias potentially associated with evaporative conditions. We find no relationship between carbon and oxygen isotope abundances in our sample set that would suggest diagenetic alteration (Supplementary Fig. 7)^58^, and no covariation between diagenetic indicators (Mn/Sr or δ^18^O) and U isotope (Supplementary Fig. 7) or Ce anomaly (Supplementary Fig. 8) values, indicating that these parameters have not been systematically altered through diagenetic processes.

### Cerium anomaly

**Fig. 3:**
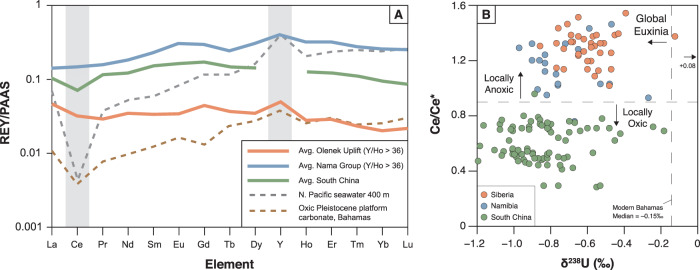
Rare-earth element and δ^238^U comparison. **A** Rare-earth element + yttrium (REY) spider plots for average Olenek Uplift samples (orange; this study), average Nama Group samples that were investigated for U isotopes (blue; ref. 18), and average South China samples that were investigated for U isotopes (green; ref. 17). REY concentrations are normalized to post-Archean Australian shale (PAAS) values. For the Olenek Uplift and Namibian sections, only those samples with Y/Ho ratios >36 were included (no Y/Ho data are available for the South China section). Oxygenated North Pacific seawater at 400 m depth^44^ is plotted for comparison, along with the shallowest depth sample with Y/Ho > 36 from Pleistocene carbonate platform cores in the Bahamas^49^. Highlighted in gray is Ce, showing the presence/absence of a negative Ce anomaly, and Y, showing the positive Y anomaly that indicates a seawater signal. **B** Cross-plot of Ce/Ce* vs. δ^238^U for samples from this study (orange), Namibia (blue; ref. 18), and South China (green; ref. 17). Modern Bahamas median δ^238^U value is from ref. 55 and oxic/anoxic Ce/Ce* cutoff value of 0.9 is from ref. 21.

REE distributions in carbonates are currently recognized as a powerful tool to evaluate seawater patterns and redox conditions in ancient depositional basins. Given their low abundance, however, careful leaching of samples is required to avoid contamination from siliciclastic components. To this end, we used a step-wise extraction technique using dilute acids, and measured Y/Ho in our leachates^21,49^ to evaluate the fidelity of the samples. The majority of our samples show Y/Ho elevated above the detrital background (>36), resulting in a positive Y anomaly and a strongly hydrogenous (seawater) REE signal (Fig. 3). Considering only samples with Y/Ho >36, Ce/Ce* values range from 0.99 to 1.55, with a median value of 1.30, and no evidence for systematic stratigraphic changes across formation boundaries (Fig. 2). No negative Ce anomalies are recorded in any of the samples from our section. This is strong evidence for deposition beneath a largely anoxic water column through the entire Olenek Uplift succession. Reference 21 suggested that positive Ce anomalies (as are recorded in many of our samples) could be indicative of manganous conditions in the water column wherein Ce is added back to sediments by sorption to Mn (oxy)hydroxides. In that study, manganous conditions were accompanied by enrichment in Mn/Fe ratios (>0.29), which are much higher than found in the current Olenek sample set (median = 0.09). This suggests that local seawater conditions in the Olenek basin were more likely ferruginous. When considering each formation separately, the Maastakh, Khatyspyt, and Turkut formations all have low median Mn/Fe ratios (<0.11), but the Mattaia Formation has an elevated median Mn/Fe ratio of 0.28. This could suggest that the Mattaia Formation was deposited under less reducing (i.e., manganous) conditions compared to the underlying Ediacaran units. Based on the profound sulfur isotope event recorded in the Khatyspyt Formation, ref. 20 suggested locally euxinic conditions at the base of the interval, and a transition to non-euxinic conditions when ‘superheavy pyrite’ appears in the stratigraphic record. Our data further constrain local Khatyspyt redox conditions, suggesting that deposition of the entire succession occurred under anoxic conditions and that the δ^34^S data may indicate a local shift from euxinic to ferruginous conditions (not discernable using Ce anomalies) or a shift in global sulfate availability. Regardless, our Ce anomaly data from Olenek Uplift carbonates strongly suggest deposition beneath an anoxic water column through the entirety of the succession.

### Uranium isotopes

On a global scale, U isotope abundances in carbonates have been used recently to assess the extent of euxinic water masses, which today only occupy around 0.05% of the ocean floor, primarily in the Black Sea, Cariaco Basin, and Norwegian fjords. Under such euxinic conditions, isotopically heavy U(IV) is preferentially removed to sediments, leaving residual seawater U(VI) enriched in the light ^235^U isotope. The median δ^238^U value for syn-depositional and post-depositional Bahamian carbonates deposited in the well-oxygenated modern oceans is −0.15‰^55^, such that lower values recorded in ancient carbonates likely indicate an expansion of global marine euxinia compared to today. In our sample set, the median δ^238^U value is −0.58‰ in the Maastakh Formation, −0.64‰ in the Khatyspyt Formation, −0.57‰ in the Turkut Formation, and −0.53‰ in the Mattaia Formation (average analytical uncertainty = ±0.08‰ 2 s.d. based on replicate measurements). The dataset as a whole has a median δ^238^U value of −0.58‰, which is significantly lower than modern carbonates (Supplementary Fig. 9), indicating widespread marine euxinia during the terminal Ediacaran Period and additionally during Cambrian Stage 2. Our Olenek δ^238^U values are similar to those recorded in terminal Ediacaran sections from Namibia (median = −0.77‰)^18^ and South China (median = −0.87‰)^17,59^ (Supplementary Fig. 10). We attribute the slightly heavier δ^238^U values in the Olenek Uplift to differences in local redox environments where Ce/Ce* data indicate that the Olenek Uplift was deposited under anoxic conditions, the Namibian sections under mixed conditions^18,21^, and the South China sections under oxic conditions^17,59^ (Fig. 3). Uranium isotopes recorded under anoxic/euxinic pore water conditions tend to be enriched in the heavier isotope by ~0.3‰ as compared to coeval seawater^55,60^, which matches the offset between the anoxic Olenek Uplift and oxic South China records. The oxic South China section would be expected to more closely capture global seawater δ^238^U compositions, and the more negative values there (down to −1.2‰) are the lightest recorded thus far in the geological archive, including those measured in multiple sections across the Permo-Triassic boundary during which anoxia is thought to be a primary driver of the largest Phanerozoic extinction event^61–65^ (Supplementary Fig. 9). These integrated observations support the view that the terminal Ediacaran oceans, where the Ediacara biotas evolved and thrived, were more reducing than at any other time in Earth history thus far investigated using U isotopes.

## Discussion

Despite our carbonate-based determinations of pervasive positive Ce anomalies indicative of anoxic depositional conditions in the Olenek basin, the Khatyspyt Formation contains a diverse array of Ediacaran macrofossils preserved in situ (Supplementary Figs. 1 and 2). This includes *Nenoxites curvus*, a rangeomorph *Charnia masoni*, an arboreomorph *Khatyspytia grandis*, macrophytes, and various holdfast structures^34^. The combination of in situ Ediacaran macrofossils and geochemical data suggesting a locally reducing water column is strong evidence that this Ediacaran assemblage was living under anoxic conditions. Whereas basal, sessile metazoans have a lower oxygen demand than large, motile animals^66,67^, all animals require some oxygen for their metabolism, with the only possible exception being meiofaunal loriciferans living permanently in the absence of molecular oxygen^68,69^, although the true oxygen demand of these organisms is still debated^70,71^. The absence of a negative Ce anomaly in Olenek basin waters suggests the lack of Mn-oxidation, which occurs in modern environments at sub-micromolar oxygen concentrations (3 μM or less)^46^. Bilaterians have been recorded in modern environments at O_2_ concentrations as low as ~1 μM, which ref. 72 suggests is near the theoretical limit needed to support these animals. It is possible, then, that members of the Khatyspyt Ediacaran assemblage were metazoans able to survive under lower O_2_ conditions than are needed to generate a negative Ce anomaly. These exceedingly low O_2_ concentrations would severely limit the size and metabolic rate of metazoans, however, which is inconsistent with the macroscopic size of the Khatyspyt fossils (although this depends on body construction and may not apply to some diploblasts). The sub-micromolar O_2_ living conditions of the Khatyspyt Ediacaran assemblage suggest instead that these organisms may not have metazoan affinities, unless they developed special adaptations to permanently anoxic conditions (e.g., the use of hydrogenosome-like organelles instead of mitochondria in their anaerobic metabolic cycle)^68^. Another intriguing possibility is that this Ediacaran assemblage lived in association with epibiotic or endosymbiotic bacteria^8,10^, by analogy with recent metazoans persisting at very low oxygen levels of only a few micromolar per kg^73–76^.

Alternatively, it is important to consider the possibility that our recorded Ce/Ce* values represent a time-averaged signal that is not responding on the same time scale as local ecological change. For example, ref. 40 reported Ediacaran body fossils and, importantly, clear bilaterian trace fossils from shales that record an anoxic iron speciation signal in northwestern Canada. Additionally, ref. 77 investigated a core through the organic-rich Cambrian Alum Shale in Bornholm, Denmark and found that the presence of benthic fauna (e.g., trilobites, brachiopods) can be tied to brief (<3000 year) episodes of seafloor oxygenation that occurred despite an overall anoxic geochemical signature recorded for this unit. It therefore possible, then, that the Khatyspyt soft-bodied Ediacara assemblage colonized the seafloor during brief periods of oxygenation in environments that were frequently impinged upon by anoxic waters. In modern systems, ecological recovery by macrofauna after hypoxic or anoxic episodes typically occurs on the order of several years^78^, and in environments with seasonal hypoxia or anoxia, metazoan colonization similarly responds on seasonal scales^79,80^. Regardless of this possibility, our data demonstrate that some members of the soft-bodied Ediacara biota were well-adapted to environments with low and fluctuating oxygen levels. The potential for physiological tolerance of frequent anoxia is supported by the suggestion that ancestral eukaryotes were facultatively anaerobic and that obligate aerobiosis is a derived trait^81,82^. If a facultatively anaerobic lifestyle was more widespread in early eukaryotes, it is reasonable that some Ediacaran organisms had greater tolerance of fluctuating redox conditions within generally low-oxygen environments.

The U isotope data from several terminal Ediacaran basins are consistent across space and time, suggesting expanded euxinia in the world oceans (most likely along productive continental margins) associated with the evolution and diversification of Ediacaran macroorganisms. Despite the spread of H_2_S-rich water masses, the Ediacara biota persisted on several continental margins up until the Ediacaran-Cambrian boundary, including a substantial diversity of Erniettomorph and Rangeomorph taxa^83^. This suggests that Ediacaran communities could have existed in oxic refugia above anoxic and potentially euxinic water masses, or that some members of the Ediacaran macrobiota were tolerant of low-oxygen conditions, or both. Our Ce/Ce* data from the Olenek Uplift clearly show a diverse assemblage of Ediacaran macroorganisms preserved in a setting that was often anoxic, thus supporting their low-oxygen tolerance.

Our data for both local and global redox conditions also shed light on the anomalous behavior of the terminal Ediacaran sulfur cycle. ^34^S-enriched pyrite (in which the sulfur isotopic composition matches or even exceeds that of carbonate-associated sulfate [CAS] or bedded sulfates) is seen in multiple Ediacaran successions worldwide, including in northwestern China^41,84^, South China^36^, Namibia^39,42^, northwestern Canada^40^, and Oman^38^. Various explanations for the ‘superheavy pyrite’ have come forth, but none of them have addressed the issue in the framework of prolonged late Ediacaran euxinia. As documented in the δ^238^U compositions of Ediacaran carbonates, the oxygenation state of the oceans oscillated between oxic and anoxic states. Following δ^238^U evidence for ocean ventilation (oxidation) during the Shuram negative δ^13^C excursion^13^, the subsequent terminal Ediacaran nadir of δ^238^U values is associated with expanded post-Shuram ocean euxinia and positive δ^13^C anomalies recorded in both South China and arctic Siberia. This coupling supports the canonical interpretation of δ^13^C fluctuations as resulting from enhanced burial and sequestration of organic matter in the global ocean^85,86^, which is promoted under conditions of expanded anoxia. This interval also reveals a profound stratigraphic step up (>40‰) in the δ^34^S signature of pyrite, with stratigraphic trends in multiple sections rising to values that meet and exceed those of seawater sulfate. Such a phenomenon requires removal of ^32^S from the oceans through the microbial production of H_2_S and its burial as insoluble pyrite in sediments when soluble iron is available. The mixing of globally expanded euxinic waters (as evidenced by the exceptionally light carbonate δ^238^U values) with ferruginous waters that may have still dominated the deep Ediacaran oceans^87^ provides a biogeochemical mechanism for the starvation of sulfate in the ocean (resulting in ^34^S enrichment of the residual mass) after the Shuram Excursion and before the Ediacaran-Cambrian boundary. These observations contrast with δ^238^U and δ^34^S evidence for greater oxygenation with higher sulfate and alkalinity in seawater associated with the earlier Shuram Excursion^13,88,89^, as well as across the subsequent Ediacaran-Cambrian transition, when bedded sulfates are recognized worldwide^38,59^. This geochemical and lithological worldview further highlights the oscillatory redox landscape of the Ediacaran Period.

The emerging picture of Ediacaran redox trends is further consistent with a spike in redox-sensitive trace metal concentrations in euxinic shales at ~560 Ma, which was interpreted by ref. 15 as an oceanic oxygenation event, although an alternative explanation involving Mn oxide shuttling has been proposed^90^. This metal enrichment event was followed by a return to crustal values in the terminal Ediacaran Period, with another oxygenation event occurring at ~540 Ma. Together, widespread oceanic euxinia in the terminal Ediacaran Period can explain: (1) positive carbonate δ^13^C values promoted by enhanced organic carbon burial; (2) anomalously light carbonate δ^238^U values; (3) ‘superheavy pyrite’ δ^34^S values caused by seawater sulfate distillation; and (4) muted trace metal enrichments in euxinic shales caused by drawdown of the oceanic metal inventory. By contrast, oxygenation during the preceding Shuram Excursion and across the subsequent Ediacaran-Cambrian boundary was accompanied by negative carbonate δ^13^C values, near-modern carbonate δ^238^U values, ^34^S-depleted pyrites, and Phanerozoic-style trace metal enrichments.

After widespread terminal Ediacaran euxinia and potential re-oxygenation across the Ediacaran-Cambrian transition associated with the accumulation of thick sulfate deposits worldwide^38^, ref. 59 found δ^238^U evidence for a second euxinic pulse in Cambrian Stage 2. In addition, ref. 91 found two pulses of expanded anoxia—one at the beginning of and one within Cambrian Stage 2 based on δ^238^U data. Our δ^238^U data from the biostratigraphically constrained Mattaia carbonate platform are consistent with the expansion of ocean euxinia at this time. In addition, our Ce/Ce* data further indicate locally anoxic water column conditions. SSFs preserved in the Mattaia Formation reveal evidence of transport and are not found in situ, possibly indicating that these animals were living in shallower shelf and shoreface environments (where a small, oxygenated layer existed above the chemocline) and then transported offshore. A shift back to a strongly euxinic ocean in Cambrian Stage 2 could have slowed the emergence of complex Cambrian life after its initial proliferation during the more oxygenated Fortunian Stage. Frequent redox oscillation has also been invoked as a driver of animal diversification beginning in Cambrian Stage 2^92^. Alternatively, the persistence of euxinia in the global ocean—as indicated by light δ^238^U values—through the studied Olenek Uplift section could indicate that redox conditions were only a minimal factor in controlling the evolution of early complex life on Earth. These results are consistent with a compilation of shale iron speciation data that suggest only limited oxygenation across the Neoproterozoic-Phanerozoic transition^93^, with more permanent ocean oxygenation perhaps delayed until the Devonian Period^94–98^.

In summary, this study combines proxy evidence for both local and global redox conditions with paleontological observations of a diverse assemblage of Ediacaran macroorganisms preserved in carbonate rocks of the Olenek Uplift of arctic Siberia. Our data indicate that: (1) a wide array of Ediacaran macroorganisms are preserved in environments that were often anoxic, and (2) the Ediacaran macrobiota persisted on multiple continental margins through a period of widespread ocean euxinia. Our δ^238^U data are comparable to other coeval terminal Ediacaran sections worldwide, indicating that ocean euxinia was more widespread than at any other interval in Earth history examined to date using U isotopes. Evidence of local anoxia recorded in strata that preserve Ediacaran organisms in situ, combined with strongly euxinic terminal Ediacaran oceans as indicated by global U isotope data, strongly suggests that some members of the Ediacaran macrobiota were tolerant of low-oxygen conditions and might have been capable of a facultatively anaerobic lifestyle. One intriguing possibility is that Ediacaran organisms used chemolithoautotrophy (e.g., sulfide oxidation) either directly or through symbionts, exploiting redox gradients at the sediment-water interface. Alternatively, these soft-bodied Ediacara organisms might have colonized the seafloor during brief oxygenation episodes not recorded by local redox proxy data. Ultimately, our combination of local and global redox data with Ediacaran fossils in the same succession offers an integrated perspective on the relationship between ocean redox and the evolution of early macroscopic life on Earth.

## Methods

### Sample collection and preparation

Samples were collected during a 2009–2010 field expedition to the Khorbusuonka and Olenek river areas of arctic Siberia. Hand samples were collected, cut, and bulk powders created in Russia before being transported to the United States. Samples were previously analyzed for a variety of geochemical proxies by refs. 20, 99, and from these preliminary data, samples were selected for this study based mostly on wt.% carbonate, with high carbonate content being essential for reliable Ce anomaly and U isotope results. 56 of the purest carbonate samples (>75 wt.%) over 497.5 m of composite section were selected for analysis in this study.

### Petrography

Thin sections were prepared by Vancouver Petrographics for a select group of samples and analyzed using a standard petrographic microscope in plane- and cross-polarized light in order to understand diagenesis. Diagenetic pathways were assessed by visual inspection of the degree of fabric retention, and petrographic characteristics were compared to geochemical data in order to interpret the diagenetic history of the succession.

### Major and trace elements

50 mL centrifuge tubes were rinsed with 6 M HCl and Milli-Q water prior to adding powdered samples. Approximately 1.5 g of sample powder was first digested by 15 mL of 1 M HNO_3_, followed by 5 mL of concentrated HNO_3_, and finally, 20 additional mL of 1 M HNO_3_. Each sample was shaken and subject to vortex mixing to ensure all powder had reacted with acid. Samples were left to sit for 24 h at room temperature to allow for maximum carbonate dissolution. Digests were centrifuged at 3800 rpm for 9 min and the supernatant was decanted from the residue. 200 to 350 mL of the supernatant was removed, placed into 10 mL centrifuge tubes, diluted to ~200 ppm calcium (Ca) with 2% HNO_3_, and analyzed for a full suite of major and trace element concentrations on a Thermo iCAP™ quadrupole inductively coupled plasma mass spectrometer (Q-ICP-MS) at Arizona State University. Typical precision is reported based on repeated analysis of simultaneously run standards, and in this study, relative % s.d. was <6% for all reported elements. Total U concentrations were also analyzed using an Element-2 ICP-MS at the University of Maryland. Analytical precision was better than 3% based on duplicate analyses.

### Rare-earth elements

Rare-earth elements were extracted using a multi-step leaching process. For calcite samples, the first 20% of moles of CaCO_3_ were dissolved with the appropriate amount of 2% HNO_3_. The insoluble residue was then separated by centrifugation and the solution decanted and disposed. The next 40% of moles of CaCO_3_ were then dissolved using the appropriate amount of 2% HNO_3_ and centrifuged again. The solution was pipetted, diluted to 10 mL with 2% HNO_3_, and shipped to Arizona State University for elemental analysis by Q-ICP-MS. For dolomite samples, a one step of digestion, which dissolves the first 20% of moles of CaMg(CO_3_)_2_ in the appropriate amount of 2% HNO_3_ was utilized. This multi-step digestion process follows the methods suggested by ref. 48 for retrieving the most seawater-like REE data. Typical precision is reported based on repeated analysis of simultaneously run standards, and in this study, relative % s.d. was better than 4% for all REEs. Associated element Y was run using a separate calibration standard. Duplicate sample analyses for Y reported an average s.d. of ± 0.24 ppm. REE concentrations were normalized to the post-Archean Australian shale (PAAS) standard^100^ and the cerium anomaly (Ce/Ce*) was calculated using: Ce/Ce* = [Ce]_SN_/([Pr]_SN_^2^/[Nd]_SN_) where SN denotes a PAAS-normalized value^48^.

### Uranium isotopes

The remaining solution that was not used for major and trace element analysis was dried down in clean Teflon beakers for U isotope analysis. An appropriate amount of Institute for Reference Materials and Measurements (IRMM) ^236^U/^233^U double-spike (0.8 mL of spike per 500 ng U) was added to each sample before U isotope column chemistry^101^. The samples were digested sequentially using reverse aqua regia, H_2_O_2_, and concentrated HNO_3_. The spiked samples were evaporated to dryness and re-dissolved in 3 M HNO_3_. Uranium was separated twice from matrix elements by ion exchange chromatography using UTEVA resin. Separated U was dissolved in 2% HNO_3_ to a concentration of 40–50 ppb and analyzed on Thermo Scientific Neptune multi-collector ICP-MS instruments at Arizona State University and the University of Maryland (see Supplementary Fig. 13 for inter-laboratory comparison). The standard solution CRM 145 (50 ppb U) was analyzed once every two samples. A secondary standard (CRM 129a) was also analyzed after every ten analyses. Sample δ^238^U values were normalized to the average of bracketing standard CRM 145. Precision of standards and duplicate analyses is reported as 2 s.d. of replicate measurements. Average precision for the sample set is ±0.08‰. Methods are adapted from ref. 53 and further details can be found in refs. 61, 101.

## Supplementary information

Supplementary Information

Description of Additional Supplementary Files

Supplementary Data 1

Supplementary Data 2

## Data Availability

All data generated in this study are available in Supplementary Data 1 and Supplementary Data 2.
