# Supplementary material for: A diverse Ediacara assemblage survived under low-oxygen conditions
Source: Nat Commun. 2022 Nov 27;13:7306. doi: 10.1038/s41467-022-35012-y (PMC9701187; doi:10.1038/s41467-022-35012-y)
Supplement: Supplementary file 1 — Supplementary Information [file 41467_2022_35012_MOESM1_ESM.pdf]

A diverse Ediacara assemblage survived under low-oxygen conditions

Lucas B. Cherry[1,2,*], Geoffrey J. Gilleaudeau[1,*], Dmitriy V. Grazhdankin[3], Stephen J. Romaniello[4], Aaron J. Martin[5], Alan J. Kaufman[6]

[1]Department of Atmospheric, Oceanic, and Earth Sciences, George Mason University, Fairfax, Virginia, USA

[2]Department of Geology, University of Maryland, College Park, Maryland, USA

[3]Trofimuk Institute of Petroleum Geology and Geophysics, Russian Academy of Sciences, Siberian Branch, Novosibirsk, Russia

[4]School of Earth and Space Exploration, Arizona State University, Tempe, Arizona, USA

[5]Division de Geociencias Aplicadas, IPICYT, CP 78216, San Luis Potosí, San Luis Potosí, Mexico

[6]Department of Geology and Earth System Science Interdisciplinary Center, University of Maryland, College Park, Maryland, USA

[*]Corresponding authors: lucasbcherry1@gmail.com and ggilleau@gmu.edu

Supplementary Information

Supplementary Figures

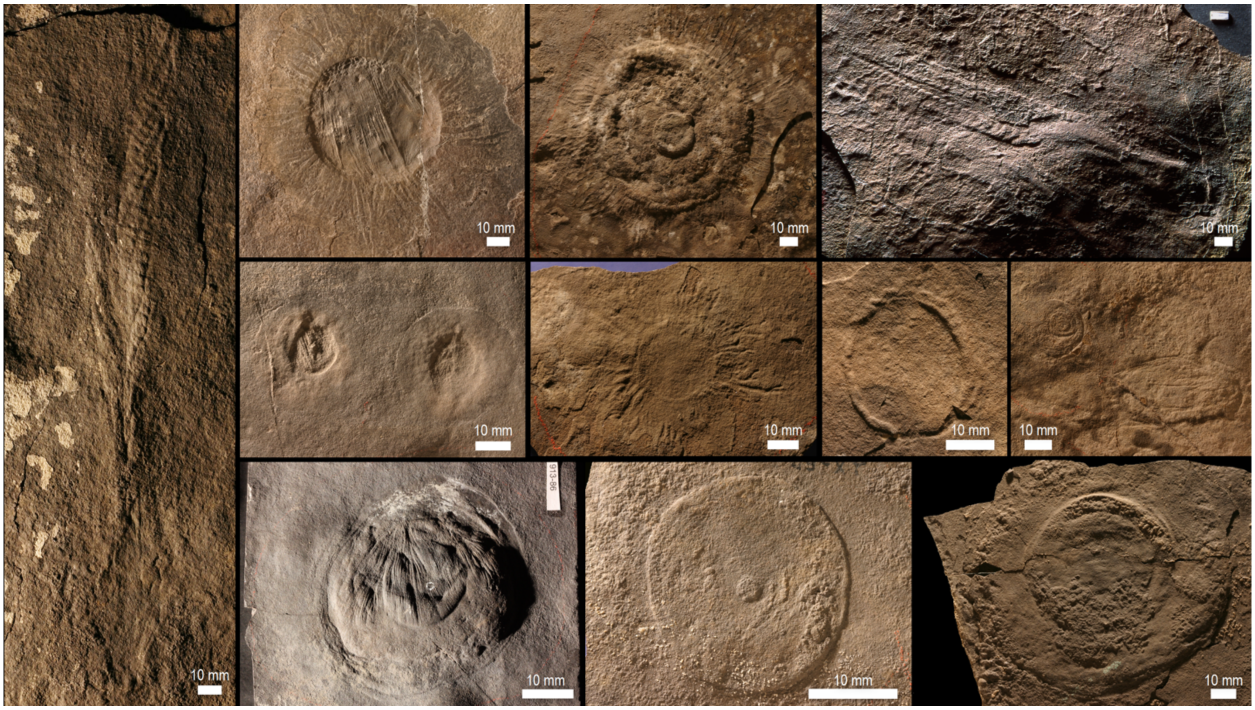

Supplementary Figure 1: Soft-bodied fossils from the Khatyspyt Formation. Various Ediacara-type soft-bodied fossils from the Khatyspyt Formation, Olenek Uplift, Siberia (see ref. [12] for full details of the Khatyspyt assemblage).

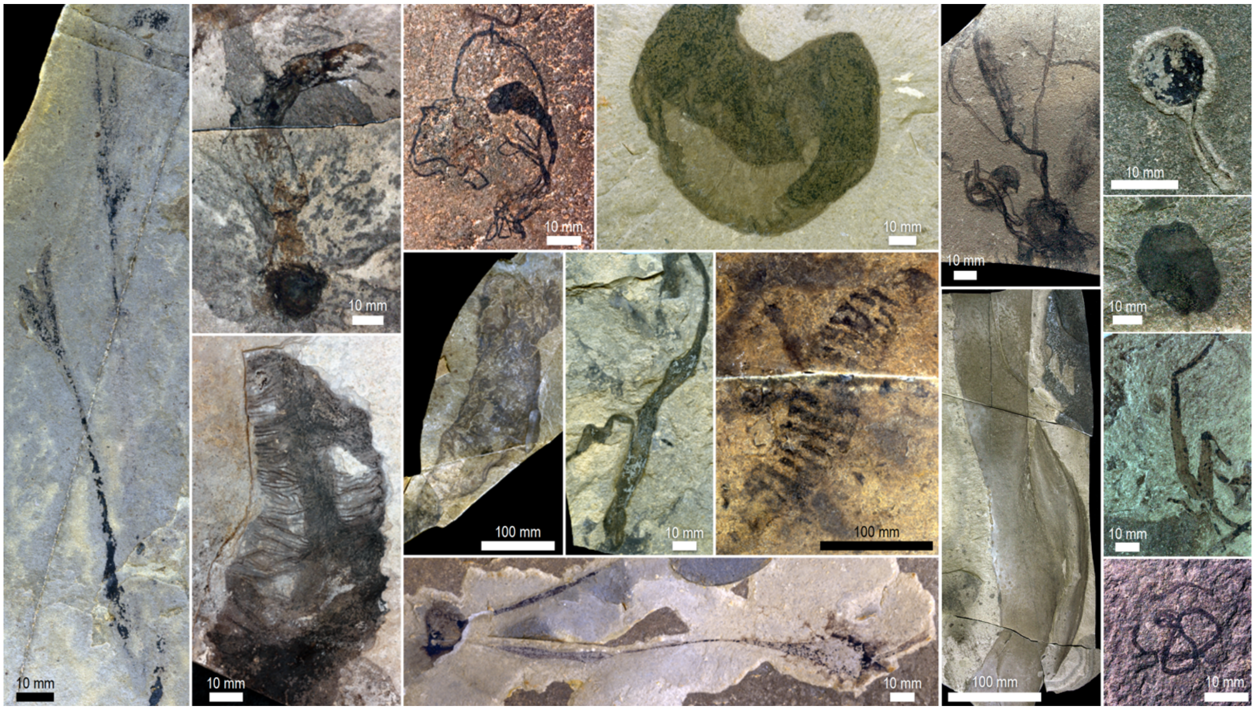

Supplementary Figure 2: Carbonaceous compressions from the Khatyspyt Formation. Various Ediacara-type carbonaceous compressions from the Khatyspyt Formation, Olenek Uplift, Siberia (see ref. [12] for full details of the Khatyspyt assemblage).

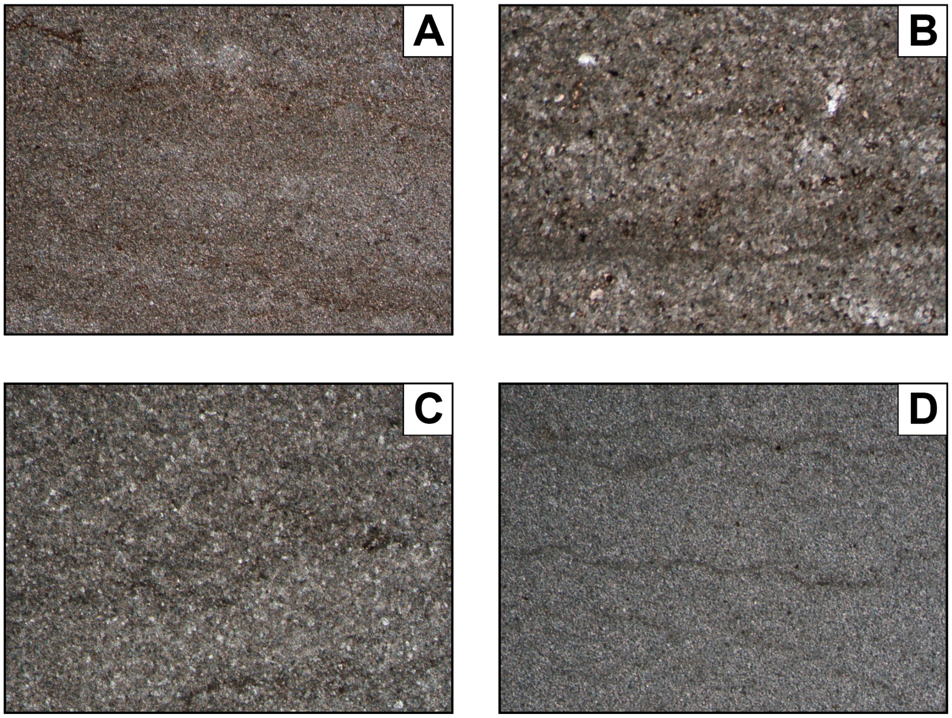

Supplementary Figure 3: Maastakh Formation photomicrographs in plane-polarized light. In panels A and D, the width of the field of view is 4.5 mm, and in panels B and C, the width of the field of view is 1.2 mm. A) Typical micritic to microsparitic fabric with thin laminations highlighted by faint wavy, brown layers (sample M601-0.5). B) Typical micritic to microsparitic fabric with thin laminations highlighted by wavy, grey to brown layers. Small, opaque peloids are also apparent (sample M601-8.5). C) Typical micritic to microsparitic fabric in an unlaminated sample without peloids (sample M601-11.5). D) Typical micritic to microsparitic fabric with thin laminations highlighted by occasional wavy, dark layers (sample M601-14.5). Overall, the micritic to microsparitic, fabric-retentive texture of dolomite from the Maastakh Formation indicates formation during early marine diagenesis and a high degree of preservation.

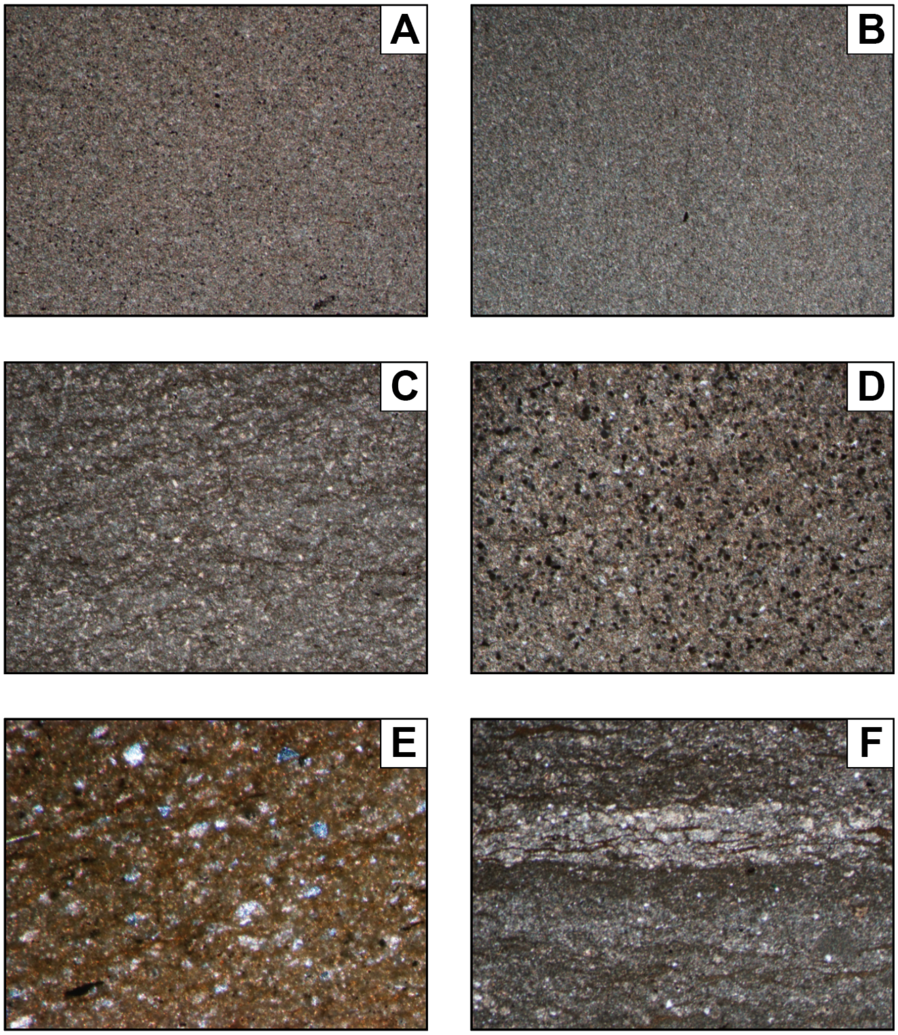

Supplementary Figure 4: Khatyspyt Formation photomicrographs. Images in plane-polarized light (panels A-D, F) and cross-polarized light (panel E). In panels A-D and F, the width of the field of view is 4.5 mm, and in panel E, the width of the field of view is 1.2 mm. A) Typical micritic to microsparitic, unlaminated fabric (sample K701-2.5). B) Homogenous micritic to microsparitic, unlaminated, well-preserved limestone (sample K701-18.2). C) Typical micritic to microsparitic fabric with abundant thin, wavy laminations (sample 1004-1060). D) Micritic to microsparitic, mostly unlaminated fabric with abundant clumps of black material which may be solid hydrocarbons (pyrobitumen) (sample 1004-1056). Specific areas containing pyrobitumen were avoided for crushing in this study. E) Thinly-laminated texture highlighted by brown organic material and fragments of quartz silt (sample K701-10.9). F) Typical fabric of the Khatyspyt Formation showing micritic to microsparitic carbonate with abundant wavy, organic-rich laminations (sample K701-10.9). Overall, the micritic to microsparitic, fabric-retentive texture of calcite from the Khatyspyt Formation indicates a high degree of preservation.

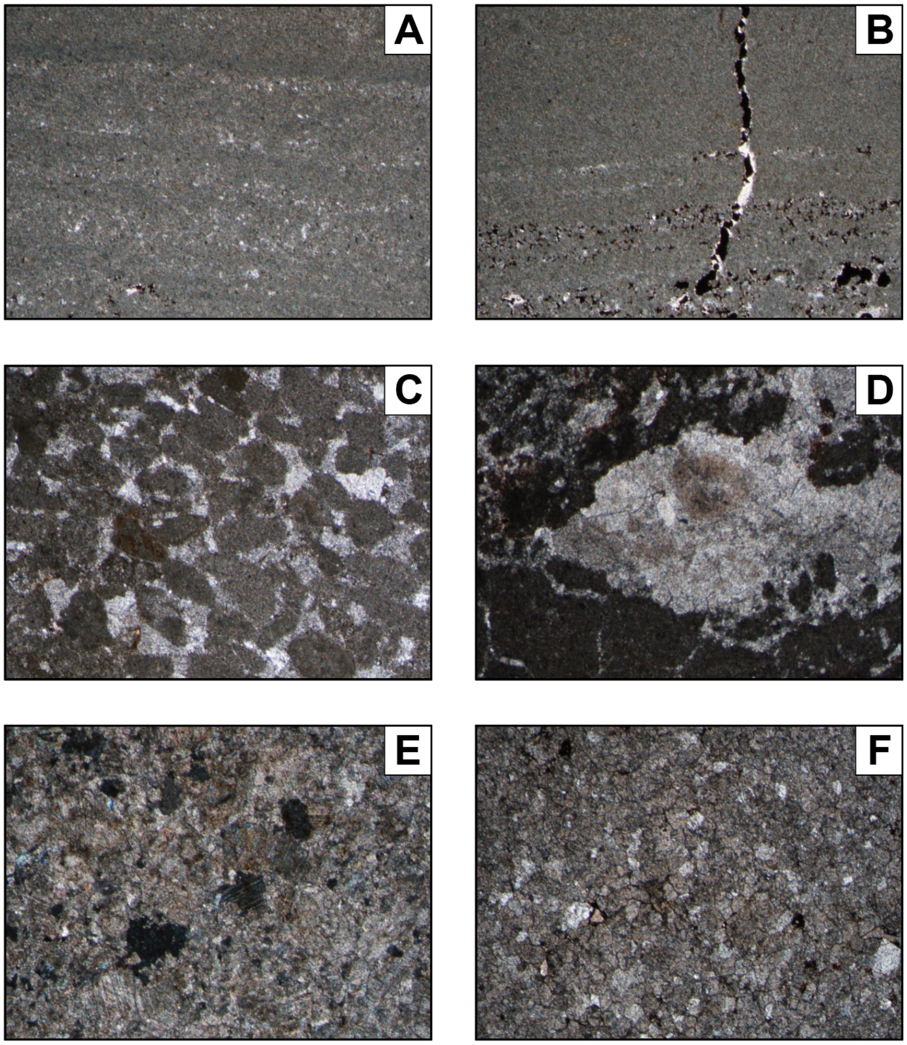

Supplementary Figure 5: Turkut Formation photomicrographs. Images in plane-polarized light (panels A-D and F) and cross-polarized light (panel E). In each photo, the width of the field of view if 4.5 mm. A) Typical micritic to microsparitic fabric of the Turkut Formation with clear sub-parallel laminations (sample T901-0.0). B) Micritic to microsparitic fabric with clear sub-parallel laminations. This photo shows opaque brown to block clumps that act to fill void space and veins. These are interpreted as migrated pyrobitumen (sample T901-0.0). Specific areas containing pyrobitumen were avoided for crushing in this study. C) Abundant subrounded to subangular intraclasts from the uppermost Turkut Formation with space between grains filled with coarse dolomite spar (sample T901-2.9). D) Complex sample from the uppermost Turkut Formation with fine-grained laminated layers disrupted by soft-sediment deformation and multiple infills of coarse-grained spar. This picture is of largest void in the sample containing coarse-grained spar (sample T901-8.3). E) Coarse-grained fabric-destructive dolomite (note the twinning in the dolomite crystals). Samples with this texture are confined to the uppermost Turkut Formation and their geochemistry is treated

with caution in this study (sample T901-14.9). F) Partially-recrystallized dolomite with brown organic material highlighting grain boundaries (sample T901-17). This fabric-destructive texture is only typical of the uppermost Turkut Formation. Overall, the majority of the Turkut Formation is reminiscent of panels A and B with a micritic to microsparitic, fabric-retentive texture indicating dolomite formation during early marine diagenesis and a high degree of preservation.

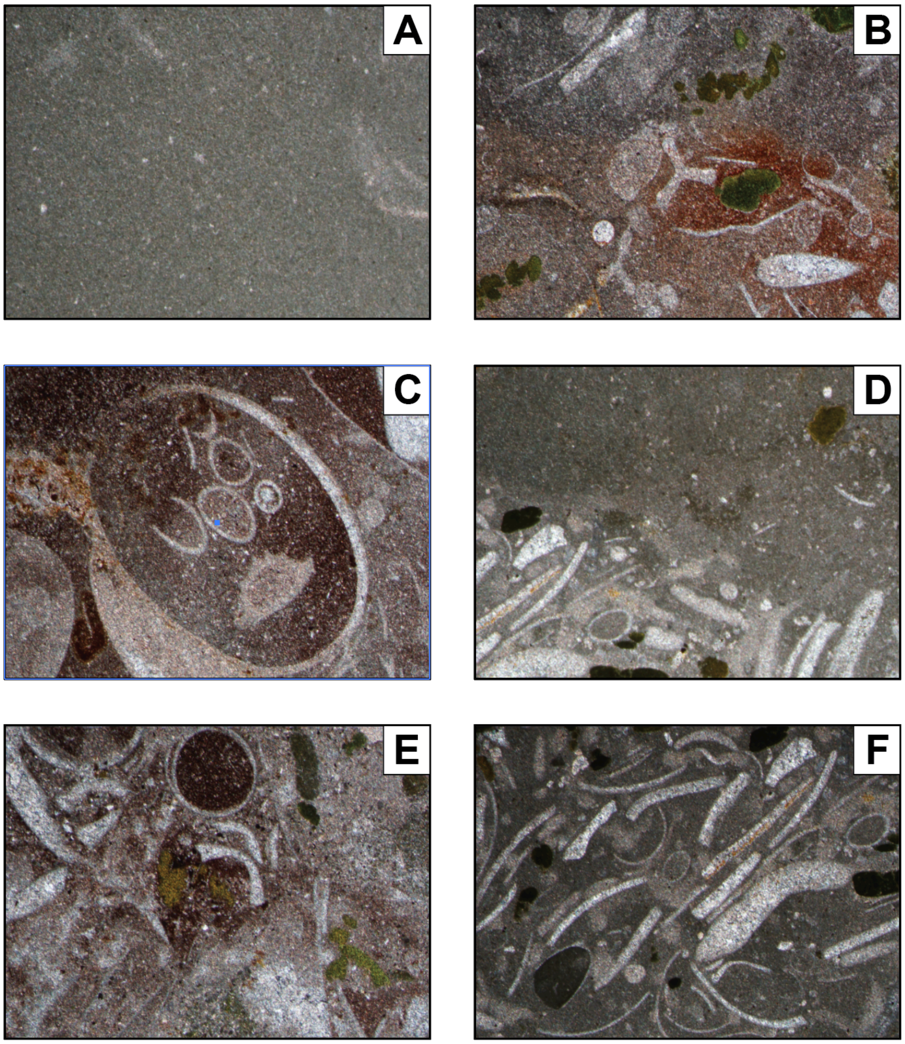

Supplementary Figure 6: Mattaia Formation photomicrographs in plane-polarized light. In each photo, the width of the field of view if 4.5 mm. A) Typical grey micritic to microsparitic calcite (sample 1002-37). B) Large, green glauconite grains and skeletal fragments within a micritic to microsparitic reddish calcite matrix (sample 1002-32). C) Reddish micritic to microsparitic calcite matrix with abundant large skeletal fragments and occasional glauconite (sample 1002-35). D) Grey micritic to microsparitic calcite matrix with skeletal fragments in discrete layers and oriented, implying transport (sample 1002-37). E) Reddish micritic to microsparitic calcite matrix with many large skeletal fragments and occasional glauconite (sample 1002-35). F) Grey micritic to microsparitic calcite matrix with large glauconite clumps and oriented skeletal fragments, implying that the skeletal fragments in the Mattaia Formation are transported and not found *in situ* (sample 1002-37). Overall, the micritic to microsparitic, fabric-retentive texture of calcite from the Mattaia Formation indicates a high degree of preservation.

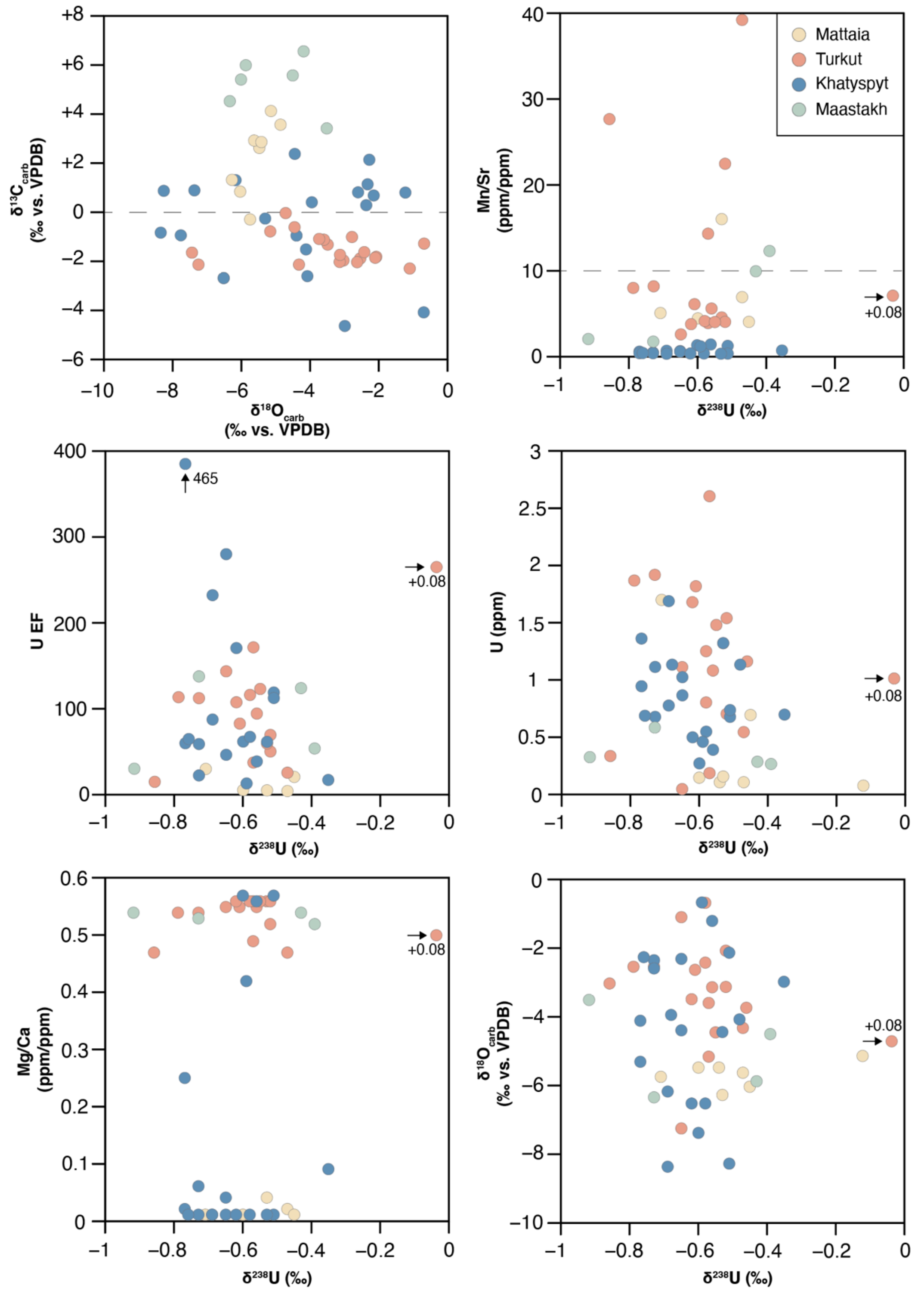

Supplementary Figure 7: Geochemical cross-plots assessing diagenetic alteration of $\delta^{238}U$ signals in our samples. Note the lack of correlation between $\delta^{13}C$ and $\delta^{18}O$, with correlation between these variables normally indicative of systematic alteration by diagenetic fluids. Mn/Sr ratios are less than the typically accepted diagenetic cutoff value of 10 in nearly all samples except several from the uppermost Turkut

Formation. There is also a lack of correlation between Mn/Sr and $\delta^{238}U$. Additionally, $\delta^{238}U$ does not correlate with uranium enrichment factor (U EF), which indicates a lack of detrital control on $\delta^{238}U$ values. This is because U EF compares the U/Al ratio of a sample with the U/Al of average crust[36], with U EF values >1 indicative of an authigenic U component from seawater above a detrital baseline. The large majority of samples in this study have U EF much greater than 1, indicating a seawater (and not detrital) $\delta^{238}U$ signal. $\delta^{238}U$ also does not correlate with U concentrations, Mg/Ca ratios, or $\delta^{18}O$, indicating a lack of systematic control of diagenetic processes and dolomitization on the $\delta^{238}U$ value of our samples.

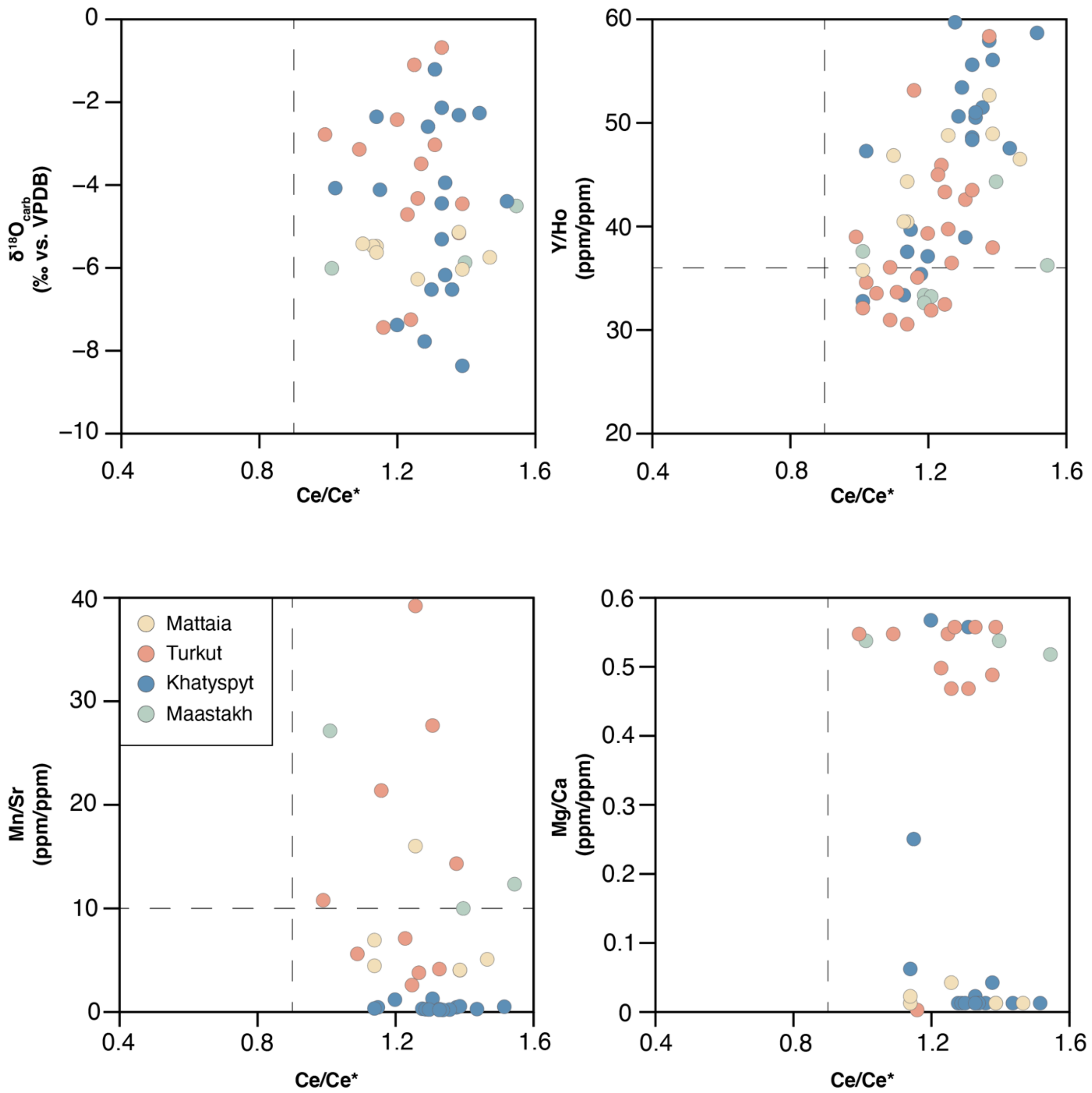

Supplementary Figure 8: Geochemical cross-plots assessing diagenetic alteration of Ce/Ce* signals in our samples. Note the lack of correlation between Ce/Ce* and typical diagenetic indicators such as $\delta^{18}O$ and Mn/Sr. Ce/Ce* also does correlate with Mg/Ca, indicating a lack of systematic alteration during dolomitization. Y/Ho ratios are used as an indicator of detrital influence on REE signals with values close to 36 (the horizontal dashed line) indicating a detrital signature[37]. It is expected that detritally-influenced samples will have Ce/Ce* close to 1; however, many of our samples have Y/Ho ratios substantially >36, indicating a seawater signature. These samples have Ce/Ce* > 1, which indicates anoxic seawater. Oxic/anoxic Ce/Ce* cutoff value of 0.9 (vertical dashed line on all plots) is from ref. [38].

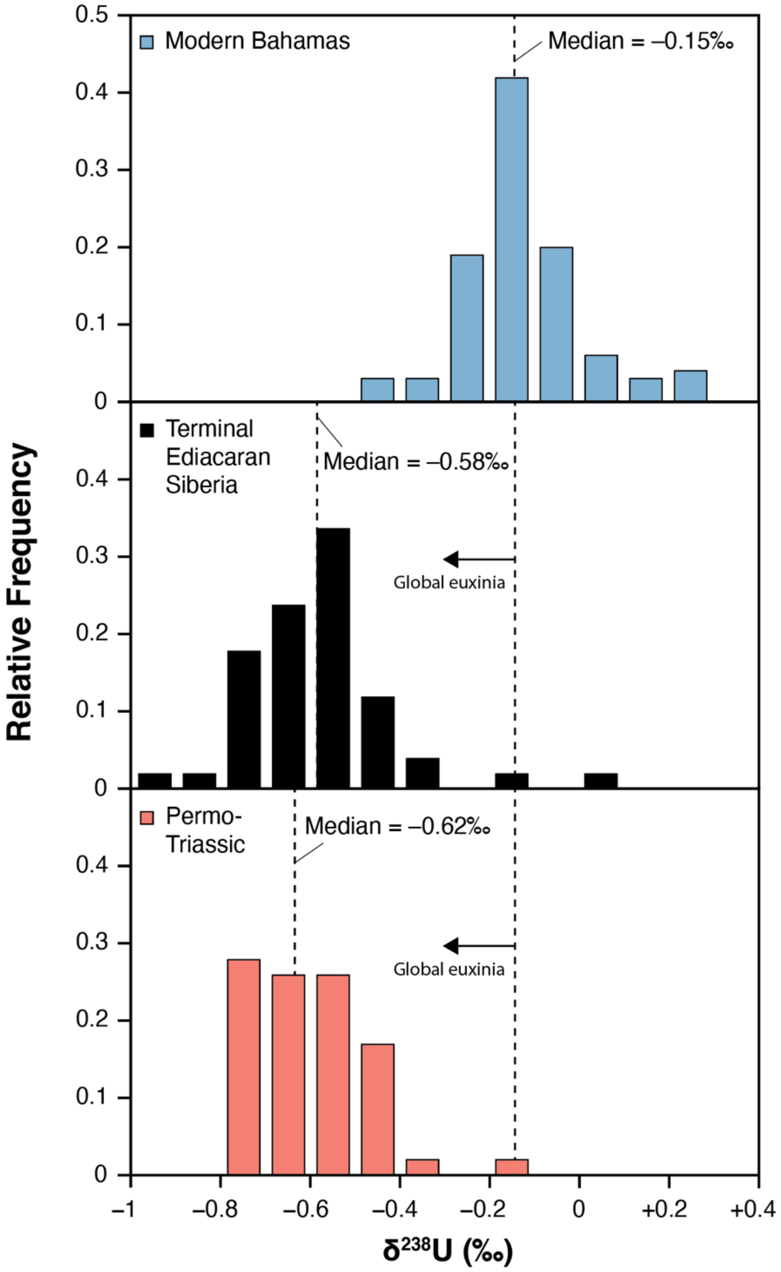

Supplementary Figure 9: Histograms showing carbonate $\delta^{238}U$ values at different times in Earth history. Modern Bahamas from ref. [39], terminal Ediacaran Siberia from this study, and Permo-Triassic from ref. [40] (also presented in ref. [34]). Note that $\delta^{238}U$ values in the terminal Ediacaran of Siberia are substantially lower than modern Bahamian carbonates, indicating expanded global ocean euxinia compared to today. Terminal Ediacaran $\delta^{238}U$ values are similar, however, to those recorded across the Permo-Triassic extinction, during which an expansion of ocean euxinia is thought to have caused the largest mass extinction event of the Phanerozoic Eon. These data highlight the expansiveness of global ocean euxinia during the terminal Ediacaran Period.

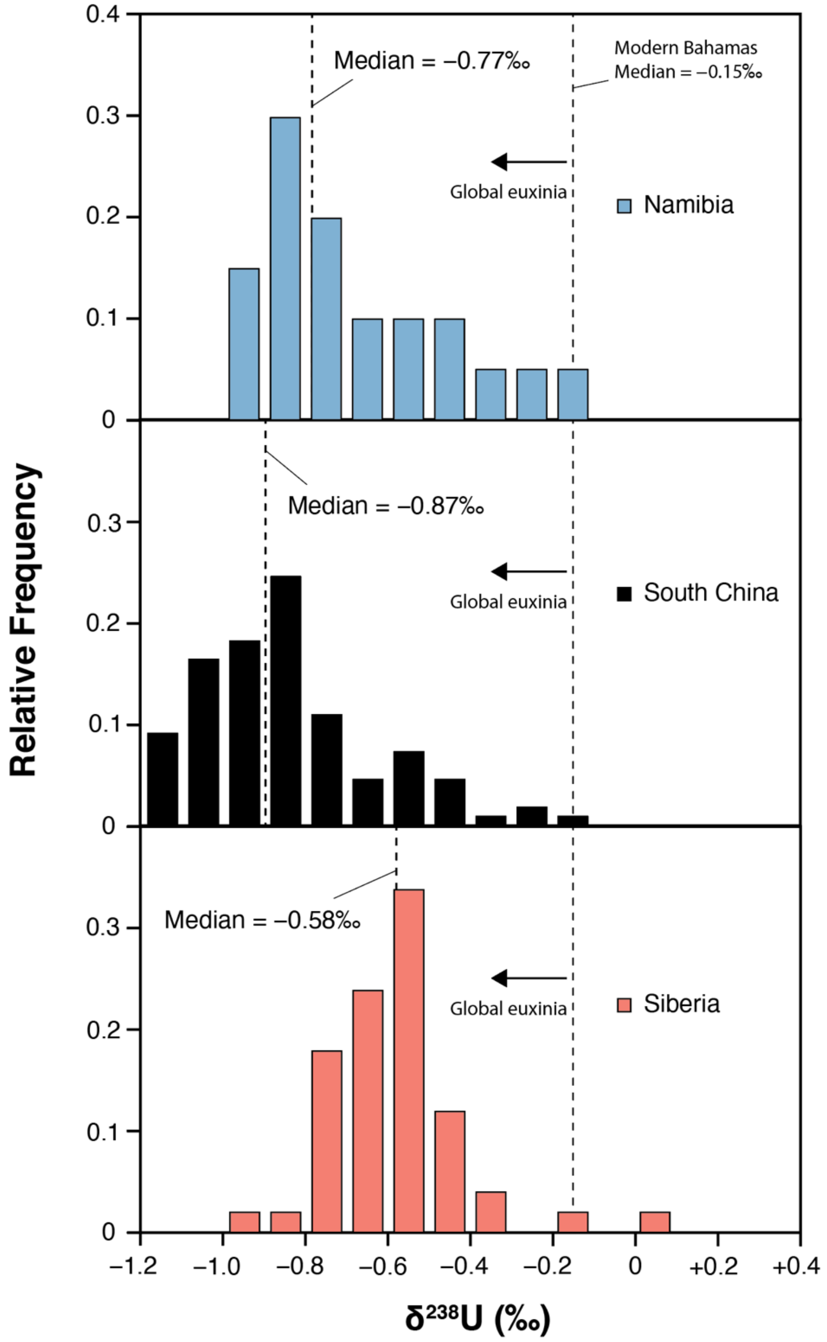

Supplementary Figure 10: Histograms showing carbonate $\delta^{238}U$ values from three main terminal Ediacaran sections discussed in the main text (Siberia, south China, and Namibia). Modern Bahamas median from ref. [39], Siberia from this study, south China from ref. [41], and Namibia from ref. [42]. Note that all sections show $\delta^{238}U$ values substantially below modern carbonates, indicating pervasive global ocean euxinia compared to today. Differences between terminal Ediacaran sections are likely related to differences in local processes during early diagenesis, as discussed at length in the main text.

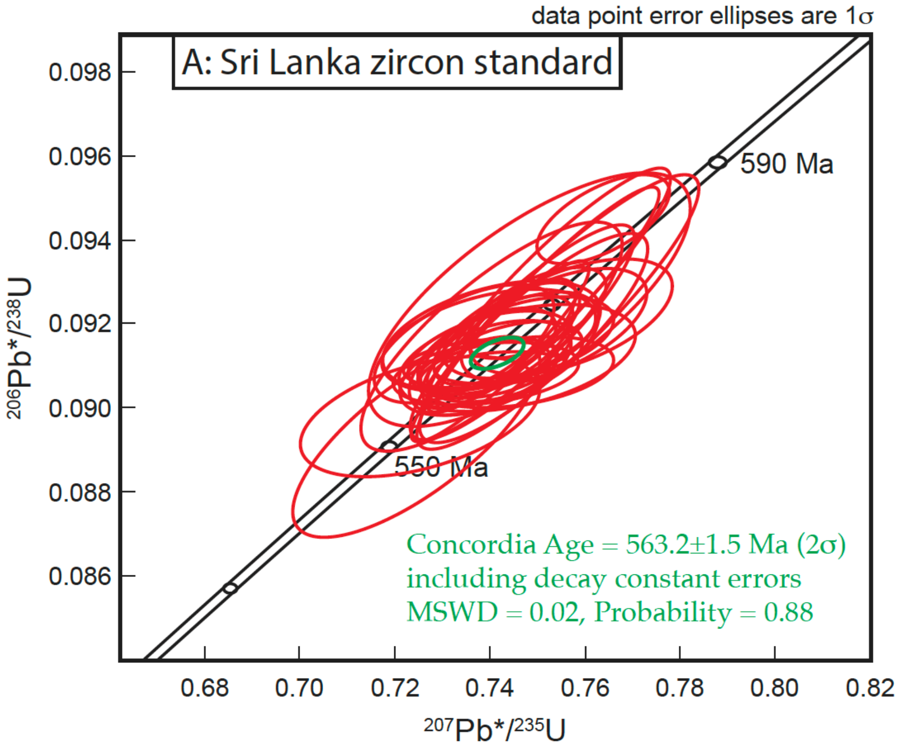

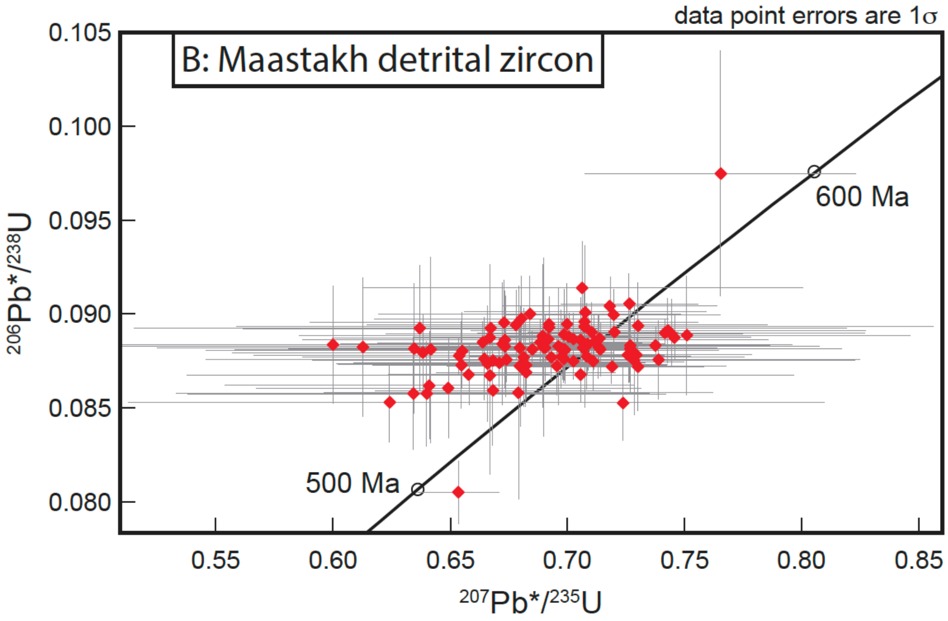

Supplementary Figure 11: Concordia diagrams for U-Pb zircon ages. (A) Concordia diagram for analyses of Sri Lanka standard zircon. (B) Concordia diagram for analyses of detrital zircon from the Maastakh Formation.

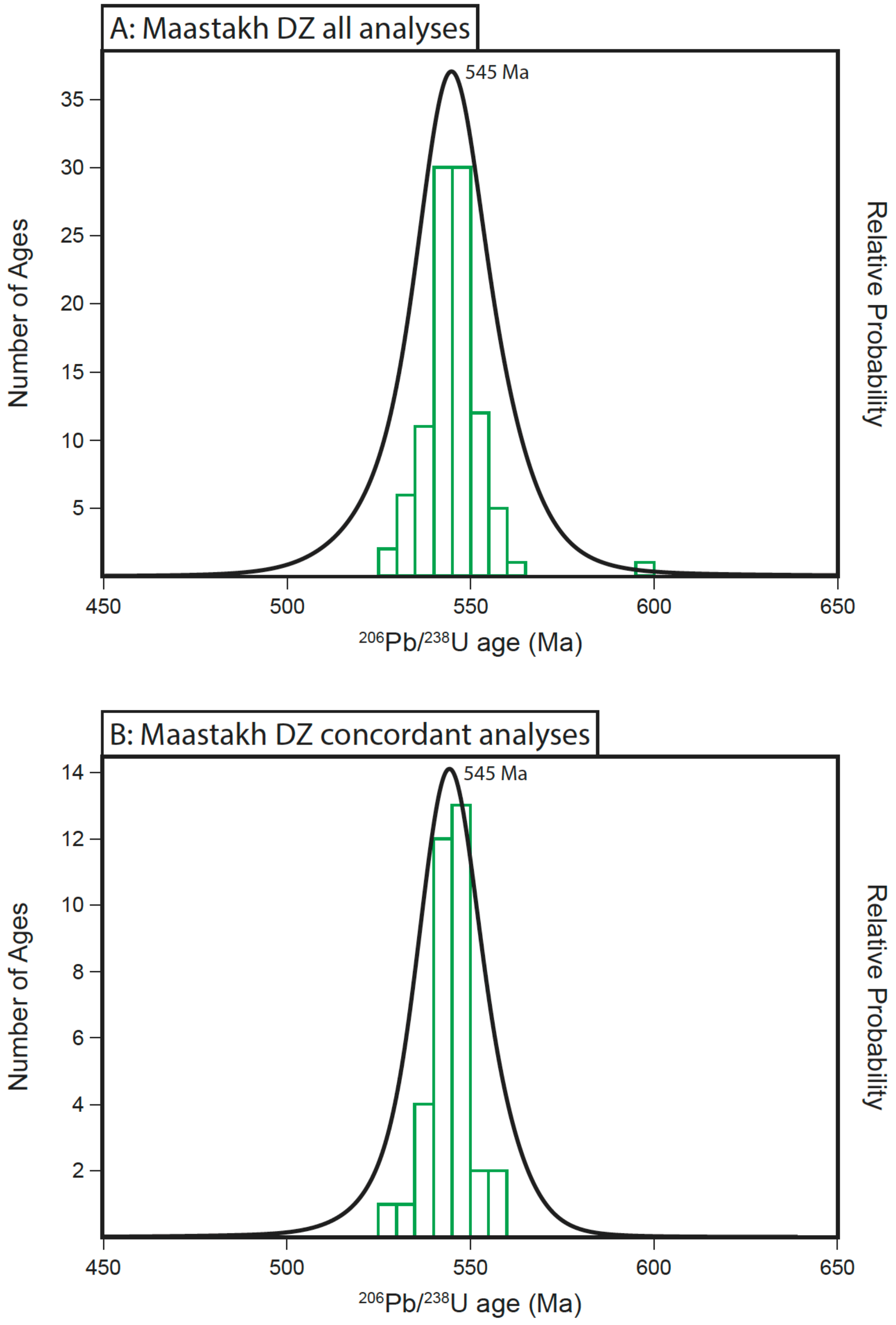

Supplementary Figure 12: Relative probability diagrams for U-Pb zircon ages. (A) Relative probability diagram and histogram for all analyses of Maastakh Formation detrital zircon except Analysis 85. (B) Relative probability diagram and histogram for 75-105% concordant analyses of Maastakh Formation detrital zircon except Analysis 85.

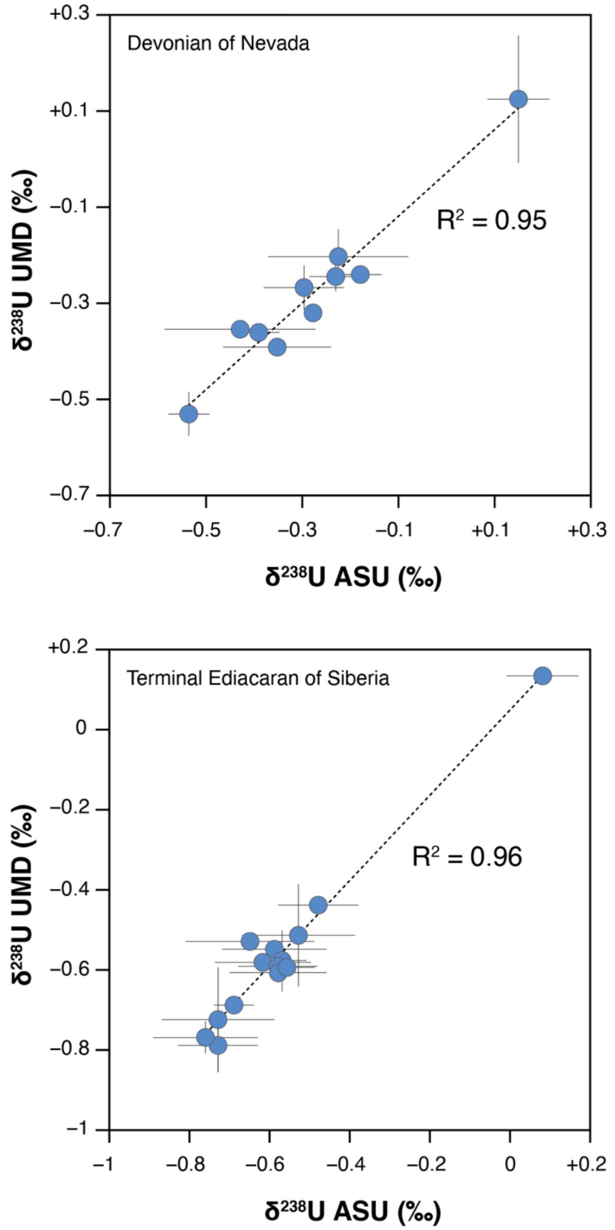

Supplementary Figure 13: Inter-laboratory δ^{238}U comparison. These cross-plots show δ^{238}U values obtained from the same ~50 ppb U solutions spiked with ^{236}U/^{233}U double-spike measured on the Neptune multi-collector ICP-MS at Arizona State University (ASU) and the Neptune Plus multi-collector ICP-MS at the University of Maryland (UMD). Devonian carbonates from Nevada where U was separated at ASU were studied[35], as well as samples from this study. Precision is reported as 2 s.d. of replicate measurements. Identical procedures were used during all analytical sessions at both institutions (see ref. [34]). The excellent agreement between the new method at UMD and the established method at ASU highlights the viability of δ^{238}U measurements at UMD in future studies.

Supplementary Discussion

Regional geology

The Olenek Uplift is a large, uplifted block of Archean and Paleoproterozoic crust located on the northeast edge of the Siberian craton south of the Lena Delta. The uplift lies within the Central Siberian Plateau, which is bordered on the west by the West Siberian basin, to the north by the Yenisei-Katanga trough, and to the south by the Central Asian and Mongol-Okhotsk fold belts, and the Baikal rift. In the east, it is bordered by the Verkhoyansk-Kolyma orogenic system. It is one of several uplifts on the Siberian craton that preserve Ediacaran-Cambrian sedimentary successions (e.g., the Anabar and Igarka uplifts), and the Olenek super-terrane – as one of the key building blocks of the Siberian craton – was involved in a number of Proterozoic and Paleozoic tectonic events, including the amalgamation and breakup of Rodinia[1]. Directly on top of the basement, a 2.2-km-thick siliciclastic-carbonate succession of Mesoproterozoic age was deposited, comprising the Sygynakhtakh (220 m), Kyutingde (400 m), Arymas (320 m), Debengde (500 m), and Khaipakh (670 m) formations (thicknesses in parentheses). The Khaipakh Formation is truncated by an angular unconformity, and overlain by the Khorbusuonka Group, consisting of the Maastakh, Khatyspyt, and Turkut formations. The enigmatic soft-bodied biota preserved in the Khatyspyt Formation broadly places this succession in the Ediacaran Period. Unconformably above these units lies the Kessyusa Group, which is comprised of the Syhargalakh, Mattaia, and Chuskuna formations of the Cambrian Fortunian and Stage 2. The Kessyusa Group is overlain by the archaeocyathid-bearing Cambrian Stage 2 Erkeket Formation[2].

The focus of this study are carbonates within the Maastakh, Khatyspyt, Turkut, and Mattaia formations. Along the Khorbusuonka River (Fig. 2) and its tributaries in northeastern Siberia, outcrops of these formations have a structural dip of 3 to 10° to the north. Because the river flows north-northwest, progressively higher stratigraphic levels are encountered downstream[2]. A series of small-displacement, east-west-oriented high-angle normal faults cut the Olenek Uplift[3]. Although fault displacement is only a few tens of meters, the low regional dip of the strata means that equivalent stratigraphic levels may have lateral surface offsets of up to several kilometers. As a result, while it is possible to obtain continuous measurements of individual formations at single locations, the section presented here is a composite pieced together from many sections over a distance of ~80 km.

Previously-published age constraints

Age constraints for these units are based on geochronology, chemostratigraphy, and biostratigraphy. Uranium-lead (U-Pb) zircon ages have been generated from a tuff breccia in the Syhargalakh Formation of the Kessyusa Group, dating it to 543.9 ± 0.24 Ma (TIMS)[4]. All formations found stratigraphically below this marker are assigned to the Ediacaran Period. In addition, a volcanic tuff in the

middle of the Mattaia Formation has been dated as 529.56 ± 0.24 Ma using U-Pb zircon geochronology (TIMS) (Kaufman et al., 2012), indicating that it coincides with the end of the Nemakit-Daldyn and the base of Tommotion (the interval now defined as Cambrian Stage 2; see ref. [6]). The carbon isotope chemostratigraphy of the Khorbusuonka Group reveals moderately positive excursions in both the Maastakh and the Khatyspyt formations, as well as negative $\delta^{13}C$ shifts in both the Khatyspyt and the uppermost Turkut formations[2,7,8]. The Turkut Formation negative carbon isotope anomaly, which is variably expressed across the basin due to regional stratigraphic truncation, could be associated with the globally-recognized BACE event or, alternatively, associated with the older A4 anomaly identified in Oman[9,10]. This geochemical anomaly notably precedes the first appearance of *Trepicthnus pedum* in the basal Mattaia Formation, the trace fossil that currently defines the onset of the Cambrian Period. Carbon isotope analysis of limestones in the Mattaia Formation record a strong positive $\delta^{13}C$ excursion, with values increasing from as low as –3.4‰ near the base, to an acme of +5.4‰ associated with the FAD of *Watsonella*, before falling back to near 0‰ at the top of the formation. A second positive $\delta^{13}C$ shift to values as high as +4.4‰ is preserved in limestone of the overlying Chuskuna Formation, although it is uncertain whether this reflects a second discrete anomaly or variation associated with a single biogeochemical event[6]. This strong upward $\delta^{13}C$ trend and variations in Mattaia and Chuskuna carbonates is consistent with those from successions containing Fortunian and basal Cambrian Stage 2 carbonates in other parts of Siberia (i.e., Sukharikha River[11]), northern Africa, and South China. In sum, the integrated chronologic constraints and integrated stratigraphic observations support the broad view that the Khorbusonka Group is terminal Ediacaran in age, while the Kessyusa Group comprises the Cambrian Fortunian and basal Stage 2 intervals.

Sedimentology, stratigraphy, and paleontology

The Ediacaran Khorbusuonka River succession consists of three carbonate-dominated formations, including, in stratigraphic order: the Maastakh, Khatyspyt, and Turkut formations. The Maastakh Formation, which is ~75 m thick in the study area, consists of partially-silicified peritidal dolostone (>34 m) sandwiched between siliciclastic units. The lower siliciclastic unit (<27 m) comprises medium- and coarse-grained, trough and tabular cross-stratified, mostly quartzitic channelized sandstones, with well-rounded quartz pebbles, and variegated brownish-green, reddish-brown, and maroon shales. The dolostones (>34 m) are microbially laminated, cross- and wavy-bedded, occasionally intraclastic, coarse-bedded, and desiccation-cracked, with black chert nodules and white “cauliflower” chert nodules. The upper siliciclastic unit (12 m) consists of a pebble-bearing, trough and tabular cross-bedded channelized sandstone, which was sampled for our detrital zircon U-Pb age study (see below), overlain by finely-laminated shale. All these features of the Maastakh Formation suggest deposition in a middle carbonate ramp with increased

coarse siliciclastic input near the base and near the top of the unit associated with shallowing of the basin. The dolostones contain stromatolites and non-diagnostic microbial structures, but no other biostratigraphically-relevant fossil remains. The shale near the top of the Maastakh Formation is capped by a stromatolitic unit that is truncated by grikes, conglomerates, and other erosional remnants indicating exposure and karstification. Subsequent flooding of the basin led to the accumulation of the Khatyspyt Formation, which broadly consists of ~245 m of thin- to medium-bedded bituminous limestone, thin shale beds, and intercalated volcanic tuff layers[12]. The lower reaches of the Khatyspyt Formation are characterized by the lowest occurrence of *Nenoxites curvus*[13], as well as thick packages of intraclastic limestone occurring in channelized bodies up to seven meters in thickness. These channelized deposits contain abundant tilted angular intraclasts and soft-sediment deformation structures that are laterally persistent over tens to hundreds of meters. The Khatyspyt Formation represents a unique lagerstätten of soft-bodied Ediacara biota comprising carbonaceous compressions preserved on bedding planes and 3D preservation of the rangeomorph *Charnia*, the holdfast structures of *Hiemalora* and *Aspidella*, as well as the arboreomorph *Khatyspytia*[12]. The limestones of the Khatyspyt Formation grade into mixed limestone and dolostone of the overlying Turkut Formation, which is ~120 m thick in the study area. The contact between the Khatyspyt and Turkut formations is gradational and interpreted as conformable based on field observations. Lithologies of the Turkut Formation include alternating levels of oolites, oncolites, microbial laminites, and stromatolites, as well as collapse breccias (potentially related to evaporite mineral dissolution) that in some instances are filled with pyrobitumen[7,14]. Rare tubular steinkerns have been extracted from the Turkut Formation, and these had previously been assigned to *Cambrotubulus*, suggesting a basal Cambrian age for the unit. However, these smooth-walled molds may be the remains of any tubular organisms, including those with organic walls. At present, we cannot confidently assign these to SSFs of Cambrian age. Like the Maastakh Formation, a sandstone at the top of the Turkut Formation contains grikes, conglomerates, and other erosional remnants indicating exposure and unconformity.

This unconformity is overlain by the Syhargalakh Formation of the Kessyusa Group. The Syhargalakh Formation consists of the tuff breccia noted above, as well as a 20+ meter-thick diamictite with a green-grey sandy to clayey calcareous matrix, including abundant cobble-to-boulder sized clasts in a weakly-stratified pile. The randomly-oriented angular and rounded stones were primarily sourced from the Turkut and underlying Khatyspyt formations, but also comprise occasional green igneous rocks locally derived from the Tas-Yuryakh Vocanic Complex. We consider the likelihood that the tuff breccia and volcanics in the diamictite were sourced from diatremes that cut through the underlying lithologies, but that the rounding, faceting, and haphazard accumulation of large sedimentary clasts of variable composition was the result of glacial transport. The Syhargalakh diamictite is capped by transgressive shale of the Mattaia Formation, which has a uniform thickness of 70-90 m (ref. [15]). Above the shale, the Mattaia

Formation is characterized by silty limestone, microcrystalline sandy limestone, finely-crystalline dolostone, siltstone, and alternating sandstone/siltstone layers (Cui et al., 2016). Carbonate-rich facies reappear in the upper part of the Mattaia Formation in the Sourdakh Member[6]. Shale and siltstones of the Mattaia Formation reveal evidence of animals that pervasively bioturbated the sediments[6]. Small shelly fossils in the lower reaches of the Mattaia Formation include elements of the *Purella antiqua Zone*, which are overlain by those of the *Nochoriocyathus sunnaginicus Zone* only a few meters higher in limestone of the Sourdakh Member, including the early molluscs *Watsonella crosbyi* and *Aldanella attelborensis*[6,15,16].

Post-depositional alteration

The Olenek Uplift area as a whole shows little evidence for regional metamorphism, and the diagenesis of individual units is discussed in detail by refs. [2,7,17], and is further discussed in the main text. There is evidence of various igneous intrusions (sills, dikes, and diatremes) that crosscut the formations in the study area, producing localized contact metamorphism. Igneous intrusions and contact metamorphism would cause isotope fractionation in the neighboring carbonate facies, but these areas have been avoided in this study. The Turkut Formation does contain migrated hydrocarbons in the form of pyrobitumen. However, we carefully pre-screened samples for pyrobitumen, and individual sample pieces and specific inter-sample regions containing pyrobitumen were avoided here. The fidelity of these units for persevering least-altered geochemical signals is also demonstrated by the carbon and sulfur isotope data, which match well with global patterns in terminal Ediacaran strata.

Detrital zircon U-Pb dating

We dated detrital zircon from a sandstone collected near the top of the Maastakh Formation to provide additional constraints on the depositional age of the Khorbusuonka Group. After disaggregation, zircon was isolated at Novosibirsk State University using gravimetric and magnetic separation techniques. U-Th-Pb isotopic data were obtained via laser ablation followed by mass spectrometry on a Nu Plasma multi-collector inductively coupled plasma mass spectrometer located in the Arizona LaserChron Center at the University of Arizona following the methods described in ref. [18]. Analyses of the Sri Lanka zircon standard (564 ± 3 Ma)[19] bracketed every five analyses of the Maastakh Formation zircon; the analyses of the Sri Lanka zircon standard were used for fractionation calibration. During our analytical session, 32 standard analyses gave a concordia age of 563 ± 2 Ma (Table S2 and Fig. S11A). Data were plotted, and weighted means calculated, using IsoPlot 4.15 (ref. [20]). We discuss all uncertainties at the 2-sigma level.

The Maastakh Formation zircon U-Th-Pb isotopic data are listed in Table S1 and shown in Fig. S1B. Analysis 85 came from a spot with much higher uranium concentration than all other spots, more than double the next-highest uranium concentration. This analysis also yielded an outlying $^{206}Pb/^{238}U$ date that

was much younger than all other $^{206}Pb/^{238}U$ dates. The remaining ninety-eight analyses yielded $^{206}Pb/^{238}U$ dates between 599 and 527 Ma. Uranium concentrations were moderate to low, 32-212 ppm, with a mean of 92 ppm. None of the analyses were more than 20% normally-discordant, but sixty-four percent of the analyses were nominally 5% or more reversely-discordant. However, nearly all of the analyses were concordant within 1-sigma measurement error (Fig. S11B). Concordant and normally-discordant analyses gave $^{206}Pb/^{238}U$ dates similar to those from the reversely-discordant analyses.

Given that damage to a zircon crystal from radioactive decay facilitates lead loss from the crystal[21,22], and higher uranium concentration leads to more radiation damage, we infer that the $^{206}Pb/^{238}U$ date from high-uranium Analysis 85 is younger than the other $^{206}Pb/^{238}U$ dates due to partial lead loss. This date does not represent the time of zircon crystallization, so we do not include it in our interpretations of maximum depositional age.

Three possible explanations are typically considered for reverse discordance of a zircon U-Pb analysis older than a few million years. (1) Geologic disturbance to the U-Pb isotopic system in the zircon, particularly redistribution of lead within the zircon crystal[22-26]. This effect has been demonstrated mostly in zircon that was highly damaged by radioactive decay and/or crystal-plastic deformation. (2) A problem with the dating method, resulting in incorrect dates. For example, invalid corrections for the isotopic fractionation generated during the analysis can produce reversely-discordant dates that do not indicate the time of zircon crystallization[27,28]. (3) Large error in the measurement of ^{207}Pb, resulting in an apparently discordant analysis because of the large errors in the isotopic ratios that involve ^{207}Pb. In this case, the $^{206}Pb/^{238}U$ date can correctly indicate the actual crystallization age of the zircon, but there is no information about concordance of the analysis.

We consider explanation 1 unlikely in our case because this effect has been demonstrated mostly in zircon that accumulated abundant damage to the crystal structure, but we do not expect abundant damage in our analyzed grains. The grains have moderate to low U concentrations, are only about 550 million years old, and the Maastakh Formation experienced only weak brittle deformation. Furthermore, the Maastakh Formation was not metamorphosed. In contrast, most published examples of disturbance to the U-Pb isotopic system in zircon come from rocks that were metamorphosed to high grades. The similarity of the $^{206}Pb/^{238}U$ dates from the reversely-discordant, concordant, and normally-discordant analyses suggests that disturbance to the U-Pb isotopic system was not responsible for the reversely-discordant analyses.

Explanation 2 likewise seems unable to explain the high degree of reverse discordance in some of our analyses because the Sri Lanka standard zircon analyses have no apparent problems, and their concordia age is identical to the published crystallization age within error. Further, the similarity of the $^{206}Pb/^{238}U$ dates from the Maastakh Formation reversely-discordant, concordant, and normally-discordant analyses suggests that analytical difficulties were not the main cause of the reverse discordance. This is because

analytical problems leading to reverse discordance should skew the $^{206}Pb/^{238}U$ dates away from the correct values, whereas the $^{206}Pb/^{238}U$ dates from the concordant and slightly-discordant analyses should not be correspondingly altered. On the other hand, we note a potential mismatch between the Sri Lanka zircon standard and the Maastakh Formation zircon. Lower U concentrations in the Maastakh zircon, combined with similar crystallization ages, imply less radiation damage to the Maastakh Formation zircon. Less radiation damage in unknown zircon as compared to the standard used for fractionation calibration can lead to shallower ablation pits in the unknown zircon and too-young U-Pb dates, although the dates are not always measurably affected[29-32]. Although we cannot rule this effect out, we note that when they occur, offsets toward younger dates are less than 10%, which equates to 5-6 million years for the Maastakh Formation zircon.

Explanation 3 appears to best account for most of the reverse discordance observed in the Maastakh Formation detrital zircon analyses. Most analyses, including the nominally reversely-discordant analyses, overlap concordia within 1-sigma measurement error (Fig. S11B). Difficulty in accurately and precisely measuring isotopic ratios involving ^{207}Pb in the Maastakh Formation zircon is unsurprising because the crystals have moderate to low uranium concentrations and are young relative to the age of Earth formation, resulting in low $^{235}U/^{238}U$ ratios at the time of zircon crystallization. Accordingly, we take the $^{206}Pb/^{238}U$ date as the best indicator of the crystallization age of each zircon grain.

Following ref. [33], we used the following three techniques to calculate a maximum depositional age for the Maastakh Formation sandstone. All exclude the outlying youngest date from Analysis 85. (1) The weighted mean of the two youngest $^{206}Pb/^{238}U$ ages plus the uncertainty on the weighted mean. (2) The youngest peak on a relative probability diagram of all the $^{206}Pb/^{238}U$ ages. (3) The youngest peak of the $^{206}Pb/^{238}U$ ages from analyses that were 75 to 105% concordant. These three methods gave identical maximum depositional ages. Our reported maximum depositional age is the result from each technique rounded up to the nearest 10-million-year increment to account for the uncertainties listed above. These include possible Pb loss, as well as the potential mismatch between the zircon standard used for the fractionation calibration and the Maastakh Formation detrital zircon.

The two youngest analyses came from spots 4 and 31, yielding $^{206}Pb/^{238}U$ dates of 527 ± 25 Ma and 528 ± 26 Ma, respectively, including both measurement and systematic errors. The weighted mean of these dates is 527 ± 18 Ma (MSWD = 0), giving 545 Ma as the weighted mean plus the uncertainty. The relative probability curves for all analyses and for only 75-105% concordant analyses are nearly identical, with a single peak at 545 Ma (Figs. S12A, S12B). Thus, all three techniques for calculating a maximum depositional age gave the same result. We round this result up, making 550 Ma our reported maximum depositional age for the Maastakh Formation sandstone.

$\delta^{238}U$ method comparison

In order to validate our measurements of δ^{238}U on the Neptune Plus multi-collector ICP-MS at the University of Maryland (UMD), we conducted an inter-laboratory comparison between UMD and established instrument protocols on the Neptune multi-collector ICP-MS at Arizona State University (ASU) (see ref. [34]) where much of the data for this study were generated. The same ~50 ppb U solutions spiked with ^{236}U/^{233}U double-spike were introduced to both instruments, and all analytical sessions at both institutions employed sample-standard bracketing with the standard solution CRM 145 (50 ppb U) analyzed once every two samples. A secondary standard (CRM 129a) was also analyzed after every ten analyses at both institutions. All sample δ^{238}U values were normalized to the average of bracketing standard CRM 145 and precision of all standards and duplicate analyses is reported as 2 s.d. of replicate measurements.

We first utilized samples of Devonian carbonate from Nevada that had previously been dissolved, digested, and subjected to ion exchange chromatography for U separation at ASU for a separate study[35]. Analyzing these samples (n = 10) at both UMD and ASU produced excellent agreement (R^2 = 0.95: see Fig. S13A). We then utilized a set of samples from this study of the Olenek Uplift in a similar fashion. Analyzing these samples (n = 14) at both UMD and ASU also produced excellent agreement (R^2 = 0.96: see Fig. S13B). Average precision for the entire Olenek Uplift sample set run at ASU is ± 0.08‰. Average precision for all samples run in replicate at UMD is ± 0.05‰. Overall, these results demonstrate the viability of δ^{238}U analysis at the University of Maryland for future studies.

Supplementary References

[1]Wingate, M.T.D., Pisarevsky, S.A., Gladkochub, D.P., Donskaya, T.V., Konstantinov, K.M., Mazukabzov, A.M., Stanevich, A.M., Geochronology and paleomagnetism of mafic igneous rocks in the Olenek Uplift, northern Siberia: Implications for Mesoproterozoic supercontinents and paleogeography. Precambrian Research 170, 256-266: doi: 10.1016/j.precamres.2009.01.004 (2009).

[2]Knoll, A.H., Grotzinger, J.P., Kaufman, A.J., Kolosov, P., Integrated approaches to terminal Proterozoic stratigraphy: an example from the Olenek Uplift, north-eastern Siberia. Precambrian Research 73, 251-270: doi: 10.1016/0301-9268(94)00081-2 (1995).

[3]Krasilshchikov A.A., Biterman I.M., Proterozoic group of the Olenek uplift Markov F.G. (Ed.), Geology of the USSR Western Part of the Yakutian ASSR, Nedra, Moscow, pp. 91-100 (in Russian) (1970).

[4]Bowring, S., Grotzinger, J., Isachsen, C., Knoll, A., Pelechaty, S., Kolosov, P., Calibrating rates of early Cambrian evolution. Science 261, 1293-1298: doi: 10.1126/science.11539488 (1993).

[5]Kaufman, A.J., Peek, S., Aaron, J., Cui, H., Grazhdankin, D., Rogov, V., Xiao, S., Buchwaldt, R., Bowring, S., A shorter fuse for the Cambrian Explosion? Geological Society of America Annual Meeting, Charlotte, North Carolina, USA (2012).

[6]Grazhdankin, D.V., Marusin, V.V., Izokh, O.P., Karlova, G.A., Kochnev, B.B., Markov, G.E., Nagovitsin, K.E., Sarsembaev, Z., Peek, S., Cui, H., Kaufman, A.J., Quo vadis, Tommotian? Geological Magazine 157, 22-34: doi: 10.1017/ S0016756819001286 (2020).

[7]Pelechaty, S.M., Kaufman, A.J., Grotzinger, J.P., Evaluation of $\delta^{13}C$ chemostratigraphy for intrabasinal correlation: Vendian strata of northeast Siberia. GSA Bulletin 108, 992-1003: doi: 10.1130/0016-7606(1996)108 <0992:EOCCFI>2.3.CO;2 (1996).

[8]Cui, H., Grazhdankin, D.V., Xiao, S., Peek, S., Rogov, V.I., Bykova, N.V., Sievers, N.E., Liu, X-M., Kaufman, A.J., Redox-dependent distribution of early macro-organisms: Evidence from the terminal Ediacaran Khatyspyt Formation in Arctic Siberia. Palaeogeography, Palaeoclimatology, Palaeoecology 461, 122-139: doi: 10.1016/j.palaeo.2016.08.015 (2016).

[9]Bowyer, F.T., Yu Zhuravlev, A., Wood, R., Shields, G.A., Zhou, Y., Curtis, A., Poulton, S.W., Condon, D.J., Yang, C., Zhu, M., Calibrating the temporal and spatial dynamics of the Ediacaran-Cambrian radiation of animals. Earth-Science Reviews 225, 103913: doi: 10.1016/ j.earscirev.2021.103913 (2022).

[10]Nelson, L.L., Ramezani, J., Almond, J.E., Darroch, S.A.F., Taylor, W.L., Brenner, D.C., Furey, R.P., Turner, M., Smith, E.F., Pushing the boundary: A calibrated Ediacaran-Cambrian stratigraphic record from the Nama Group in northwestern Republic of South Africa. Earth and Planetary Science Letters 580, 117396: doi: 10.1016/j.epsl.2022.117396 (2022).

[11]Kouchinsky, A., Bengston, S., Pavlov, V., Runnegar, B., Torssander, P., Young, E., Ziegler, K., Carbon isotopes stratigraphy of the Precambrian-Cambrian Sukharikha River section, northwestern Siberian platform. Geological Magazine 144, 609-618: doi: 10.1017/S0016756807003354 (2007).

[12]Grazhdankin, D.V., Balthasar, U., Nagovitsin, K.E., Kochnev, B.B., Carbonate-hosted Avalon-type fossils in arctic Siberia. Geology 36, 803-806. doi: 10.1130/g24946a.1 (2008).

[13]Rogov, V., Marusin, V., Bykova, N., Goy, Y., Nagovitsin, K., Kochnev, B., Karlova, G., Grazhdankin, D., The oldest evidence of bioturbation on Earth. Geology 40, 395-398: doi: 10.1130/g32807.1 (2012).

[14]Bartley, J.K., Pope, M., Knoll, A.H., Semikhatov, M.A., Petrov, P.Y., A Vendian–Cambrian boundary succession from the northwestern margin of the Siberian Platform: stratigraphy, palaeontology, chemostratigraphy and correlation. Geological Magazine 135, 473-494: doi: 10.1017/s0016756898008772 (1998).

[15]Nagovitsin, K.E., Rogov, V.I., Marusin, V.V., Karlova, G.A., Kolesnikov, A.V., Bykova, N.V., Grazhdankin, D.V., Revised Neoproterozoic and Terreneuvian stratigraphy of the Lena-Anabar Basin and north-western slope of the Olenek Uplift, Siberian Platform. Precambrian Research 270, 226-245: doi: 10.1016/j.precamres.2015.09.012 (2015).

[16]Parkhaev, P.Y., Karlova, G.A., Taxonomic revision and evolution of Cambrian mollusks of the genus Aldanella Vostokova, 1962 (Gastropoda: Archaeobranchia). Paleontological Journal 45, 1145-1205: doi: 10.1134/s0031030111100030 (2011).

[17]Peek, S., Geochemical and radiometric constraints on the redox history of late Ediacaran oceans. MS Thesis, University of Maryland (2012).

[18]Gehrels, G., Pecha, M., Detrital zircon U-Pb geochronology and Hf isotope geochemistry of Paleozoic and Triassic passive margin strata of western North America. Geosphere 10, 49-65: doi: 10.1130/GES00889.1 (2014).

[19]Gehrels, G.E., Valencia, V.A., Ruiz, J., Enhanced precision, accuracy, efficiency, and spatial resolution of U-Pb ages by laser ablation-multicollector-inductively coupled plasma-mass spectrometry. Geochemistry, Geophysics, Geosystems 9, Q03017: doi: 10.1029/2007GC001805 (2008).

[20]Ludwig, K.R., User's manual for Isoplot 3.70. Berkeley Geochronology Center Special Publication 4, 76 p. (2008).

[21]Utsunomiya, S., Palenik, C.S., Valley, J.W., Cavosie, A.J., Wilde, S.A., Ewing, R.C., Nanoscale occurrence of Pb in an Archean zircon. Geochimica et Cosmochimica Acta 68, 4679-4686: doi: 10.1016/j.gca.2004.04.018 (2004).

[22]Pidgeon, R.T., Nemchin, A.A., Cliff, J., Interaction of weathering solutions with oxygen and U-Pb isotopic systems of radiation-damaged zircon from an Archean granite, Darling Range Batholith, Western Australia. Contributions to Mineralogy and Petrology 166, 511-523: doi: 10.1007/s00410-013-0888-z (2013).

[23]Mattinson, J.M., Graubard, C.M., Parkinson, D.L., McClelland, W.C., U-Pb reverse discordance in zircons: the role of fine-scale oscillatory zoning and sub-micron transport of Pb. *In* Basu, A., Hart, S. eds., Earth Processes: Reading the Isotopic Code, Washington, D. C., American Geophysical Union, Geophysical Monograph Series 95, 355-370: doi: 10.1029/GM095p0355 (1996).

[24]Romer, R.L., Alpha-recoil in U-Pb geochronology: effective sample size matters. Contributions to Mineralogy and Petrology 145, 481-491: doi: 10.1007/s00410-003-0463-0 (2003).

[25]Kusiak, M.A., Whitehouse, M.J., Wilde, S.A., Nemchin, A.A., Clark, C., Mobilization of radiogenic Pb in zircon revealed by ion imaging: implications for early Earth geochronology. Geology 41, 291-294: doi: 10.1130/G33920.1 (2013).

[26]Wiemer, D., Allen, C.M., Murphy, D.T., Kinaev, I., Effects of thermal annealing and chemical abrasion on ca. 3.5 Ga metamict zircon and evidence for natural reverse discordance: insights for U-Pb LA-ICP-MS dating. Chemical Geology 466, 85-302: doi: 10.1016/j.chemgeo.2017.06.019 (2017).

[27]Wiedenbeck, M., An example of reverse discordance during ion microprobe zircon dating: an artifact of enhanced ion yields from a radiogenic labile Pb. Chemical Geology 125, 197-218: doi: 10.1016/0009-2541(95)00072-T (1995).

[28]Tichomirowa, M., Whitehouse, M.J., Nasdala, L., Resorption, growth, solid state recrystallisation, and annealing of granulite facies zircon—a case study from the Central Erzgebirge, Bohemian Massif. Lithos 82, 25-50: doi: 10.1016/j.lithos.2004.12.005 (2005).

[29]Allen, C.M., Campbell, I.H., Identification and elimination of a matrix-induced systematic error in LA-ICP-MS $^{206}Pb/^{238}U$ dating of zircon. Chemical Geology 32-333, 157-165: doi: 10.1016/j.chemgeo.2012.09.038 (2012).

[30]Steely, A.N., Hourigan, J.K., Juel, E., Discrete multi-pulse laser ablation depth profiling with a single-collector ICP-MS: sub-micron U-Pb geochronology of zircon and the effect of radiation damage on depth-dependent fractionation. Chemical Geology 372, 92-108: doi: 10.1016/j.chemgeo.2014.02.021 (2014).

[31]Solari, L.A., Ortega-Obregon, C., Bernal, J.P., U-Pb zircon geochronology by LA-ICP-MS combined with thermal annealing: achievements in precision and accuracy on dating standard and unknown samples. Chemical Geology 414, 109-123: doi: 10.1016/j.chemgeo.2015.09.008 (2015).

[32]Sliwinski, J.T., Guillong, M., Liebske, C., Dunkl, I., von Quadt, A., Bachmann, O., Improved accuracy of LA-ICP-MS U-Pb ages of Cenozoic zircons by alpha dose correction. Chemical Geology 472, 8-21: doi: 10.1016/j.chemgeo.2017.09.014 (2017).

[33]Dickinson, W.R., Gehrels, G.E., Use of U-Pb ages of detrital zircons to infer maximum depositional ages of strata: a test against a Colorado Plateau Mesozoic database. Earth and Planetary Science Letters 288, 115-125: doi: 10.1016/j.epsl.2009.09.013 (2009).

[34]Gilleaudeau, G.J., Romaniello, S.J., Luo, G., Kaufman, A.J., Zhang, F., Klaebe, R.M., Kah, L.C., Azmy, K., Bartley, J.K., Zheng, W., Knoll, A.H., Anbar, A.D., Uranium isotope evidence for limited euxinia in mid-Proterozoic oceans. Earth and Planetary Science Letters 521, 150-157: doi: 10.1016/j.epsl.2019.06.012 (2019).

[35]Elrick, M., Gilleaudeau, G.J., Romaniello, S.J., Algeo, T.J., Morford, J., Sabbatino, M., Goepfert, T.J., Chernyavskiy, P., Cleal, C., Cascales-Miñana, B., Major Early-Middle Devonian oceanic oxygenation linked to early land plant evolution detected using high-resolution U isotopes of marine limestones. Earth and Planetary Science Letters 581, 117410: doi: 10.1016/j.epsl.2022.117410 (2022).

[36]Taylor, S.R., McLennan, S.M., The geochemical evolution of the continental crust. Reviews of Geophysics 33, 241: doi:10.1029/95rg00262 (1995).

[37]Tostevin, R., Wood, R.A., Shields, G.A., Poulton, S.W., Guilbaud, R., Bowyer, F., Penny, A.M., He, T., Curtis, K.H., Clarkson, M.O., Low-oxygen waters limited habitable space for early animals. Nature Communications 7: doi:10.1038/ncomms12818 (2016).

[38]Tostevin, R., Shields, G.A., Tarbuck, G.M., He, T., Clarkson, M.O., Wood, R.A., Effective use of cerium anomalies as a redox proxy in carbonate-dominated marine settings. Chemical Geology 438, 146-162: doi: 10.1016/j.chemgeo.2016.06.027 (2016).

[39]Chen, X., Romaniello, S.J., Herrmann, A.D., Hardisty, D., Gill, B.C., Anbar, A.D., Diagenetic effects on uranium isotope fractionation in carbonate sediments from the Bahamas. Geochimica et Cosmochimica Acta 237, 294-311: doi: 10.1016/j.gca.2018.06.026 (2018).

[40]Zhang, F., Algeo, T.J., Romaniello, S.J., Cui, Y., Zhao, L., Chen, Z., Anbar, A.D., Congruent Permian-Triassic $\delta^{238}U$ records at Panthalassic and Tethyan sites: Confirmation of global-oceanic anoxia and validation of the U-isotope paleoredox proxy. Geology 46, 327-330: doi: 10.1130/g39695.1 (2018).

[41]Zhang, F., Xiao, S., Kendall, B., Romaniello, S.J., Cui, H., Meyer, M., Gilleaudeau, G.J., Kaufman, A.J., Anbar, A.D., Extensive marine anoxia during the terminal Ediacaran Period. Science Advances 4, 1-11: doi: 10.1126/sciadv.aan8983 (2018).

[42]Tostevin, R., Clarkson, M.O., Gangl, S., Shields, G.A., Wood, R.A., Bowyer, F., Penny, A.M., Stirling, C.H., Uranium isotope evidence for an expansion of anoxia in terminal Ediacaran oceans. Earth and Planetary Science Letters 506, 104-112: doi: 10.1016/j.epsl.2018.10.045 (2019).
