# Supplementary material for: A diverse Ediacara assemblage survived under low-oxygen conditions
Source: Nat Commun. 2022 Nov 27;13:7306. doi: 10.1038/s41467-022-35012-y (PMC9701187; doi:10.1038/s41467-022-35012-y)
Supplement: Supplementary file 2 — Description of Additional Supplementary Files [file 41467_2022_35012_MOESM2_ESM.pdf]

File name: Supplementary Data 1

Description: Compilation of all geochemical data presented for Olenek Uplift carbonates in this study.

File name: Supplementary Data 2

Description: U-Th-Pb isotopic data from detrital zircon of the Maastakh Formation.
